# Data on microplastic contamination of the Baltic Sea bottom sediment samples in 2015–2016

**DOI:** 10.1016/j.dib.2019.104887

**Published:** 2019-11-26

**Authors:** Elena Esiukova, Mikhail Zobkov, Irina Chubarenko

**Affiliations:** a Shirshov Institute of Oceanology, Russian Academy of Sciences, 36, Nahimovskiy Prospekt, Moscow, 117997, Russia; b Northern Water Problems Institute of the Karelian Research Centre of the Russian Academy of Sciences, 50, A.Nevskogo Prospekt, Petrozavodsk, Karelia, 185030, Russia

**Keywords:** Microplastics, Bottom, Sediments, Contamination, Modified NOAA method, μ-Raman spectroscopy

## Abstract

The contamination by microplastics particles (MPs, 0.2–5 mm) in bottom sediments of the Baltic Sea is quantified. In total, 53 sediment samples were obtained in 8 cruises of research vessels in July–October 2015 and March–December 2016. The depths from 3 to 215 m in the Gotland, Gdansk, and Bornholm Basins are covered. Primary data is provided, along with exhaustive information on sampling dates and coordinates, depths, sampling methods, extracting procedures, control measures, detection techniques, and verification by μ-Raman spectroscopy. Number of pieces per kg dry weight is determined separately for fibres, films, and fragments. Distributions by size, plastic colour, and plastic type are presented. Modified NOAA method and μ-Raman spectroscopy were applied to obtain the data, thus they can be used for comparative analyses.

**Table:** Specifications Table

Subject	Environmental Science, Ecology
Specific subject area	Microplastic Contamination, Environment
Type of data	Table Chart Graph Figure
How data were acquired	Van Veen and “Ocean-50” grab samplers, hand-operated drag; NOAA extraction; Stereomicroscope (Micromed MC2 Zoom Digital); Raman Centaur U (LTD “NanoScanTechnology”, Russia) spectrometer.
Data format	Raw and Analysed.
Parameters for data collection	Sampling of bottom sediments. Microplastics extraction according to the modified NOAA method [[1], [2], [3]]. Contamination control. Microscopy and μ-Raman spectroscopy analyses.
Description of data collection	Data of number of pieces per kg dry weight (0.2–5 mm, MPs) in bottom sediments from 3 to 215 m depth on the base of 53 samples obtained in 8 cruises of research vessels in the Baltic Sea in 2015–2016. Map of study area and sampling stations. Distribution of MPs by size and by colour. Raw μ-Raman spectroscopy intensity.
Data source location	The Baltic Sea (Gotland, Gdansk and Bornholm basins), 53 stations. Locations of the 53 station are on URL: http://lamp.ocean.ru/index.php/2016/11/18/samples-map/
Data accessibility	All data is accessible within this article.

**Table:** 

**Value of the Data** • Microplastic contamination in bottom sediments of the Baltic Sea Proper in 2015–2016 is documented. • A benchmark for future studies dedicated to microplastic contamination in the Baltic Sea is presented. • Obtained data can be used for comparative analysis of plastic contamination in bottom sediments of other seas and oceans. • Number of pieces per kg dry weight, spatial distribution, types of particles (fibres, films, fragments), as well as size, colour, and plastic types are reported.

## 1 Data

**Fig. 1:**
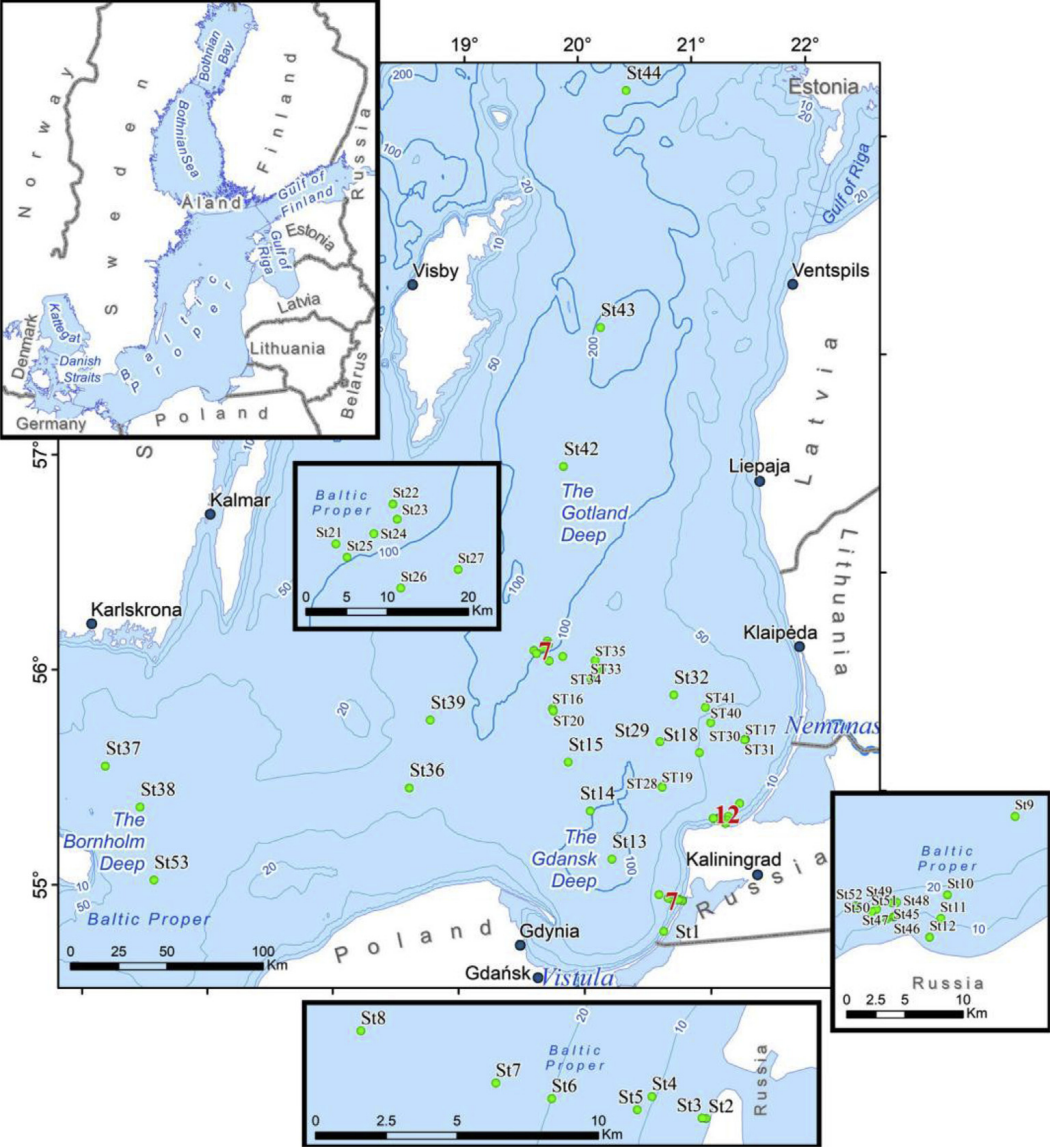
Study area and sampling stations*. * The maps were prepared with ArcGis 10.2.2 software, Natural Earth and HELCOM MADS spatial data. Depth contours from Ref. [4].

**Fig. 2:**
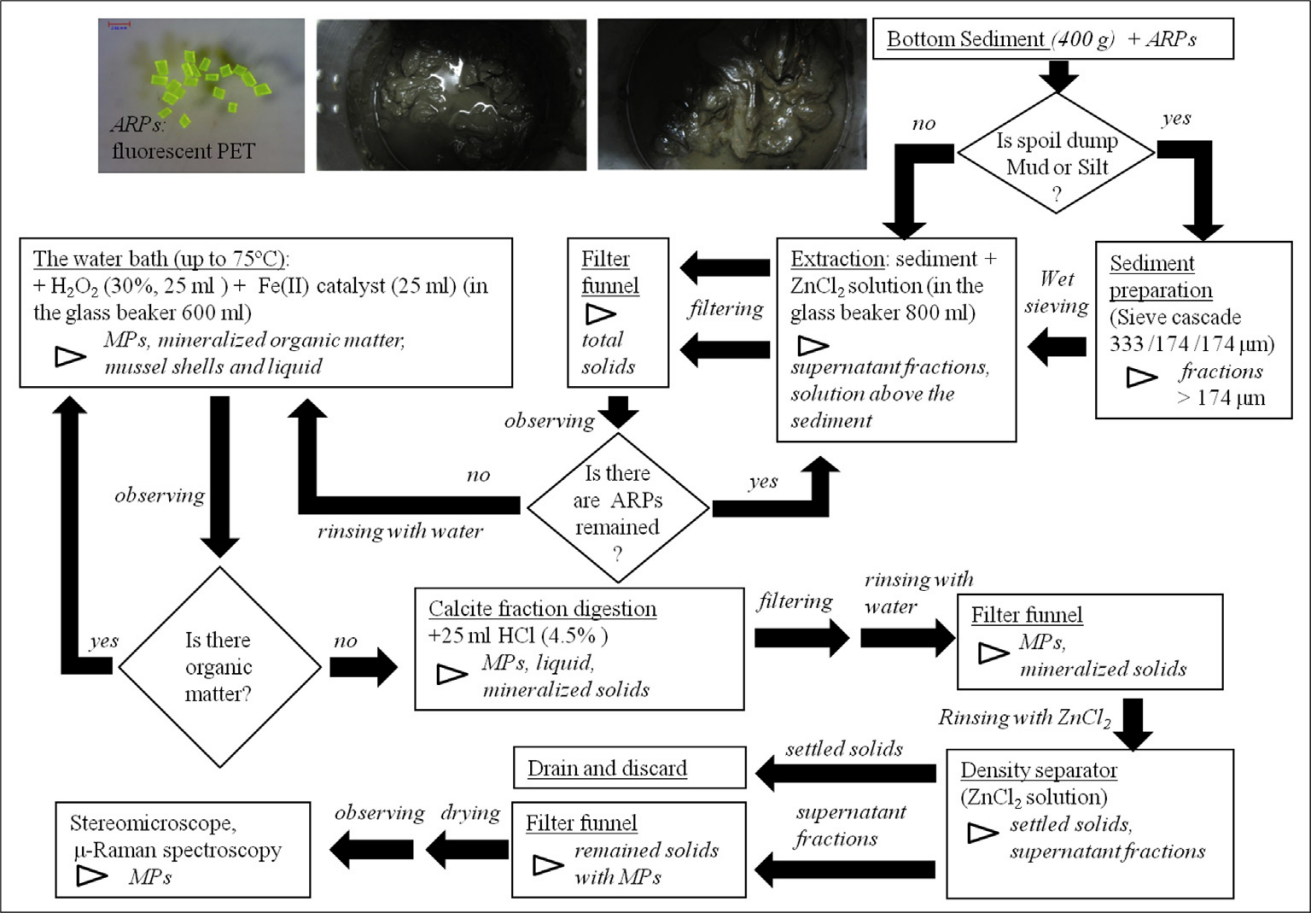
Analysis procedures: the modified NOAA method.

**Fig. 3:**
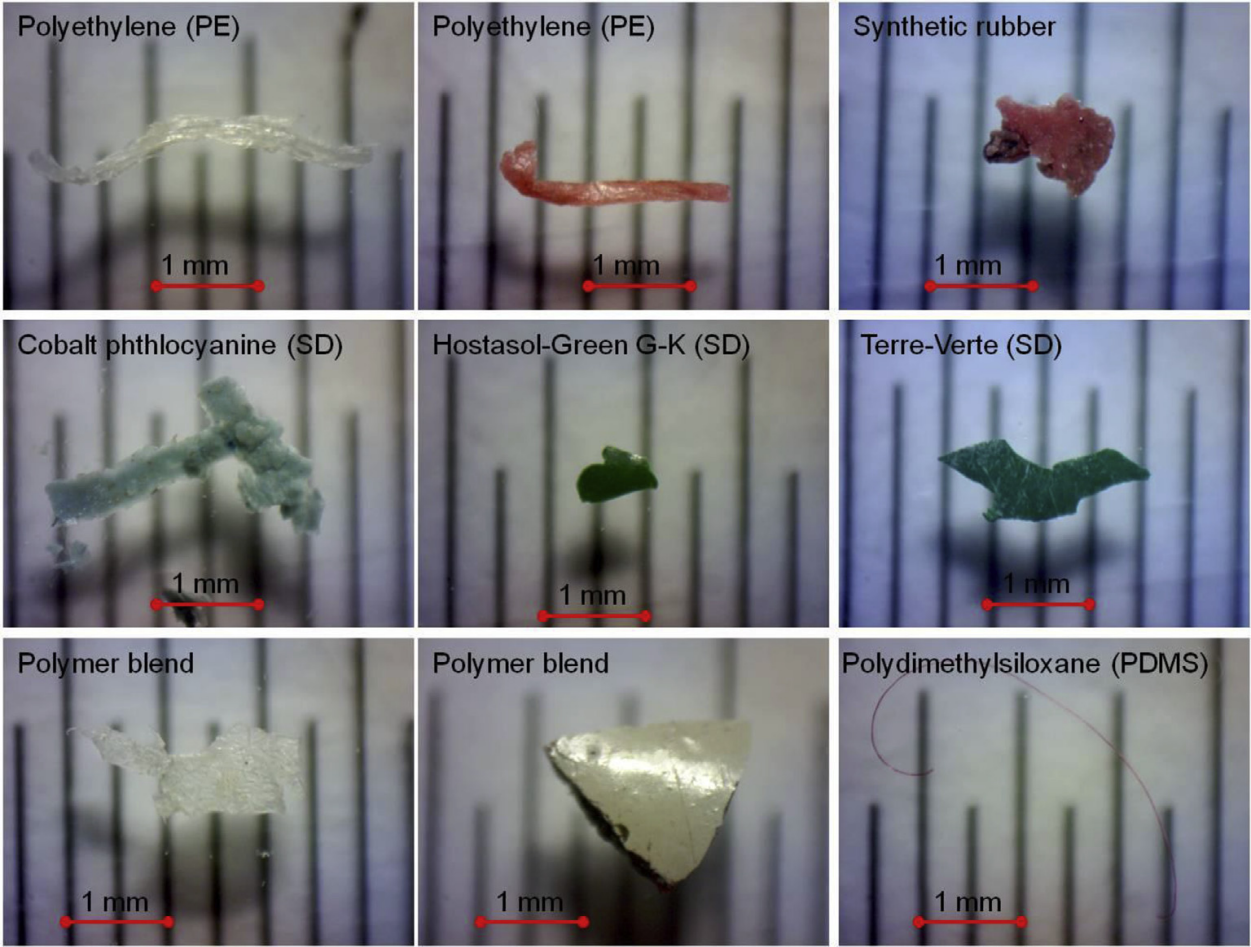
Polymer samples.

**Fig. 4:**
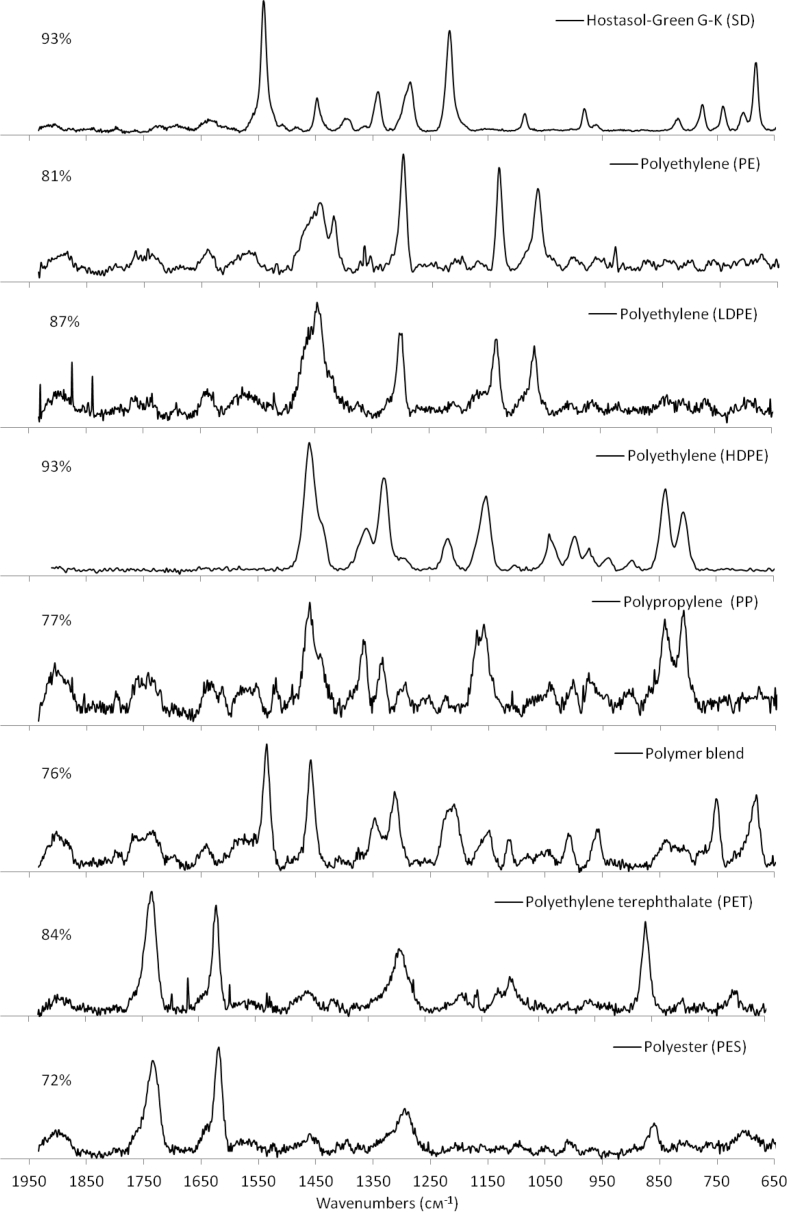
Spectra of typical MPs identified by μ-Raman spectroscopy, the hit ratio between the specimen spectra and reference spectra (in percentages).

The dataset contains information on microplastics (MPs, 0.2–5 mm) content in 53 bottom sediment samples collected in 8 cruises of research vessels in the Gotland, Gdansk and Bornholm basins of the Baltic Sea in July–October 2015 and March–December 2016. Sampling sites (Fig. 1), their geographic coordinates, sample masses, and sediment types (Table 1) are presented. MPs content is provided in total number of pieces (fibres, films, and fragments) in a sample, and in pieces per kg dry weight (pcs per kg DW) (Table 2). Laboratory analysis workflow is described (Fig. 2). Photos of nine selected MPs specimens extracted from sediments are presented (Fig. 3). Polymer types were identified by the μ-Raman spectroscopy (Table 3). The Raman spectra of typical MPs are characterized by the hit ratio to a certain polymer type (Fig. 4).

**Table 1:** Expedition data, sampling sites locations and sediment characteristics.

№ sampling stations	Vessel-station	Date	Latitude	Longitude	Depth, m	Sampler	Sampling square, m^2^	Mass of sample WW, kg	Sediment type
1	boat-9	July 3, 2015	54.50019	19.68639	9	hand-operated drag		6.7	Mixed medium to coarse sand
2	boat-3	October 9, 2015	54.62983	19.86867	3	hand-operated drag		14.5	Mixed medium or coarse sand
3	boat-5	October 9, 2015	54.63017	19.86653	5	hand-operated drag		11.8	Fine sand
4	boat-10	October 9, 2015	54.63905	19.841	10	hand-operated drag		12.5	Mixed medium or coarse sand
5	boat-15	October 9, 2015	54.63557	19.832	15	hand-operated drag		8.1	Fine sand
6	boat-20	October 9, 2015	54.64275	19.78637	20	hand-operated drag		10.1	Sand, gravel, stones
7	boat-25	October 9, 2015	54.65007	19.7572	25	hand-operated drag		6.5	Fine sand
8	boat-30	October 9, 2015	54.67225	19.6877	30	hand-operated drag		10.4	Mixed medium or coarse sand
9	NORD-1	March 30, 2016	55.04077	20.4379	32.6	Van Veen Grab	0.4	14.4	Fine sand
10	NORD-2	March 30, 2016	54.98825	20.33235	25	Van Veen Grab	0.4	11.3	Fine sand
11	NORD-3	March 30, 2016	54.97098	20.31907	18	Van Veen Grab	0.2	19.3	Mixed medium or coarse sand
12	NORD-4	March 30, 2016	54.95788	20.30043	11.5	Van Veen Grab	0.7	18.3	Fine sand
13	Sht 131-001	March 31, 2016	54.86476	19.34937	109	“Ocean-50” Grab	0.375	8.4	Mud
14	Sht 131-002	March 31, 2016	55.10067	19.22613	101	“Ocean-50” Grab	0.1875	6.7	Clayey mud
15	Sht 131-003	March 31, 2016	55.33858	19.09974	81	“Ocean-50” Grab	0.2	11.5	Clayey mud
16	Sht 131-004	March 31, 2016	55.59533	19.02877	87.5	“Ocean-50” Grab	0.125	3.5	Mud
17	Sht 131-006	April 1, 2016	55.33148	20.56187	31	“Ocean-50” Grab	0.25	16.9	Fine sand
18	Sht 131-007	April 1, 2016	55.30043	20.17428	47	“Ocean-50” Grab	0.25	1.6	Sand, gravel, stones
19	Sht 131-008	April 1, 2016	55.16738	19.83263	68	“Ocean-50” Grab	0.1	2.2	Mud
20	Sht 131-010	April 2, 2016	55.5834	19.0338	81	Van Veen Grab	0.1	10.5	Fine silty mud
21	Sht 131-011	April 2, 2016	55.87502	18.93692	106	“Ocean-50” Grab	0.25	11.1	Mud
22	Sht 131-013	April 5, 2016	55.91075	19.05808	109	“Ocean-50” Grab	0.125	2.2	Mud
23	Sht 131-014	April 5, 2016	55.89347	19.06263	109	“Ocean-50” Grab	0.125	1.3	Mud
24	Sht 131-015	April 5, 2016	55.88102	19.0135	102	“Ocean-50” Grab	0.125	3.4	Mud
25	Sht 131-016	April 5, 2016	55.85885	18.95523	104	“Ocean-50” Grab	0.2	4.0	Fine sand
26	Sht 131-020	April 5, 2016	55.81765	19.0521	95	“Ocean-50” Grab	0.125	1.8	Fine sand
27	Sht 131-021	April 5, 2016	55.83003	19.16835	75.7	“Ocean-50” Grab	0.25	3.9	Fine sand
28	Sht 132-002	June 12, 2016	55.1665	19.833	67	Van Veen Grab	0.05	6.9	Fine silty mud
29	Sht 132-003	June 12, 2016	55.37683	19.867	91	Van Veen Grab	0.05	7.2	Mud
30	Sht 132-005	June 13, 2016	55.33017	20.55817	29	Van Veen Grab	0.04	2.3	Fine sand
31	Sht 132-005	June 13, 2016	55.32983	20.557	29	Van Veen Grab	0.04	2.2	Fine sand
32	Sht 132-008	June 13, 2016	55.5835	20.03383	75	Van Veen Grab	0.04	3.8	Fine silty mud
33	Sht 132-014	June 15, 2016	55.71117	19.37483	72.5	“Ocean-50” Grab	0.125	11.1	Fine sand
34	Sht 132-016	June 15, 2016	55.74633	19.46883	71.4	“Ocean-50” Grab		0.4	Sand, gravel, stones
35	Sht 132-017	June 15, 2016	55.7915	19.43067	67.5	“Ocean-50” Grab	0.1	1.0	Fine sand
36	ANS 32-061	August 5, 2016	55.30675	17.78327	77	Van Veen Grab	0.05	1.2	Fine sand
37	ANS 32-107	August 7, 2016	55.53618	15.3192	74	Van Veen Grab	0.1	5.1	Mud
38	ANS 32-108	August 7, 2016	55.33367	15.57745	94	Van Veen Grab	0.1	3.5	Mud
39	ANS 32-203	August 14, 2016	55.60955	18.0173	67	Van Veen Grab	0.1	3.1	Fine silty mud
40	ANS 32-208	August 26, 2016	55.43155	20.30083	42.2	Van Veen Grab	0.1	0.6	Fine sand
41	ANS 32-211	August 29, 2016	55.50555	20.27662	50	Van Veen Grab	0.1	7.6	Mixed medium or coarse sand
42	ANS 32-227	September 7, 2016	56.70733	19.38575	117	“Ocean-50” Grab	0.125	1.2	Mixed medium or coarse sand
43	ANS 32-242	September 8, 2016	57.32478	19.86292	215	“Ocean-50” Grab	0.125	7.3	Clayey mud
44	ANS 32-284	September 10, 2016	58.4011	20.38942	120	“Ocean-50” Grab	0.125	4.9	Mud
45	NORD-5	October 27, 2016	54.97673	20.24697	20.7	Van Veen Grab	0.1	13.8	Mixed medium or coarse sand
46	NORD-6	October 27, 2016	54.97745	20.25677	20.5	Van Veen Grab	0.2	15.7	Mixed medium or coarse sand
47	NORD-7	October 27, 2016	54.97768	20.25875	19.8	Van Veen Grab	0.1	12.0	Coarse silt
48	NORD-8	October 27, 2016	54.98823	20.26327	24	Van Veen Grab	0.2	16.1	Mixed medium or coarse sand
49	NORD-9	October 27, 2016	54.98827	20.25722	19.5	Van Veen Grab	0.2	16.6	Mixed medium or coarse sand
50	NORD-10	October 27, 2016	54.98512	20.23522	23	Van Veen Grab	0.2	17.6	Mixed medium or coarse sand
51	NORD-11	October 27, 2016	54.98363	20.22802	22.8	Van Veen Grab	0.2	13.9	Mixed medium or coarse sand
52	NORD-12	October 27, 2016	54.99	20.20848	25.1	Van Veen Grab	0.5	30.9	Mixed medium or coarse sand
53	ANS 33-060	December 24, 2016	54.99017	15.64217	83	“Ocean-50” Grab		70.6	Mud

**Table 2:** Number of pieces (fibres, films, and fragments) in sample and per kg dry weight (pcs per kg DW).

№ sampling stations	Mass of analysed sample, g	Fragments, pcs	Films, pcs	Fibres, pcs	Cfragments, pcs per kg DW	Cfilms, pcs per kg DW	Cfibres, pcs per kg DW	Ctotal, pcs per kg DW
1	300	6	12	88	20	40	293	354
2	400	0	4	29	0	13	91	103
3	400	5	71	85	16	222	265	503
4	400	7	25	24	20	71	68	158
5	400	0	37	56	0	114	172	286
6	300	11	6	209	37	20	698	754
7	300	3	35	79	10	117	264	390
8	400	2	11	37	6	33	111	150
9	400	0	138	119	0	627	541	1168
10	400	8	85	225	30	339	897	1266
11	400	1	28	36	3	88	113	204
12	400	4	92	52	13	339	189	541
13	400	1	15	143	10	146	1396	1553
14	400	11	8	66	148	108	887	1142
15	400	4	21	80	20	104	395	519
16	400	16	12	107	155	116	1037	1308
17	400	30	10	33	115	38	127	281
18	400	58	28	53	192	93	175	460
19	400	18	10	253	88	49	1231	1367
20	400	3	45	465	60	893	9226	10179
21	400	1	100	97	8	772	748	1528
22	400	3	2	138	31	21	1426	1477
23	400	3	12	176	12	48	710	770
24	400	1	16	228	12	147	2050	2209
25	400	0	7	125	0	38	682	721
26	400	3	2	79	12	8	311	331
27	400	18	27	156	64	97	558	719
28	400	2	17	72	17	142	599	758
29	400	3	12	43	28	130	487	646
30	400	4	9	44	12	27	134	173
31	400	4	17	35	11	48	99	158
32	400	2	7	35	29	103	515	647
33	400	7	23	77	24	80	268	373
34	185	2	23	51	21	243	539	804
35	400	19	10	91	173	91	827	1091
36	400	12	9	68	40	30	228	299
37	400	10	16	90	95	152	857	1104
38	400	0	6	117	0	69	1354	1424
39	400	6	16	181	38	101	1143	1282
40	400	9	5	37	30	17	124	171
41	400	1	16	44	3	49	135	187
42	400	3	9	60	12	35	232	278
43	400	1	1	33	11	11	371	393
44	400	17	9	259	176	93	2675	2943
45	400	3	14	18	9	43	55	107
46	400	6	60	38	19	185	117	321
47	400	19	60	149	85	273	682	1040
48	400	0	30	31	0	93	96	188
49	400	0	17	30	0	50	88	138
50	400	4	23	32	12	70	97	179
51	400	4	37	47	13	116	147	276
52	400	2	20	37	7	63	116	186
53	400	26	7	54	252	63	522	837

**Table 3:** Polymer type and types of synthetic dyes identified using μ-Raman spectroscopy.

	Polymer type	Acronym	%	Types of Synthetic Dyes (SD):
1	Synthetic dyes	SD	47.2	Hostasol-Green G-K
2	Polyethylene	PE/HDPE/LDPE	11.1	Irgazin Blue
3	Polypropylene	PP	8.3	Cobalt phthlocyanine
4	Polymer blend	Polymer blend	5.6	Terrae-Verte
5	Polyethylene terephthalate/Polyester	PET/PES	4.6	Toloudine red
6	Polydimethylsiloxane	PDMS	3.7	Molybdenum oxide
7	Cellulose/Cellulose acetate	CE/CA	3.7	Titanium dioxide
8	Polyvinyl chloride	PVC	2.8	Cobalt sulphate
9	Synthetic rubber	Synthetic rubber	1.9	Motoperm Blue
10	Polystyrene	PS	0.9	Naples Yellow
11	Methyl vinyl ether	PVME	0.9	
12	Carbon	Carbon	0.9	
13	Polymer methylpentene	PMP	0.9	
14	Plasticine	Plasticine	0.9	
15	Nylon	Nylon	0.9	
16	Polytetrafluoethylene	PTFE	0.9	
17	Polyviniledene	PVDF	0.9	
18	Poly (methyl 2-methylpropenoate)	PMMA	0.9	
19	Polymethacrylamide	PMAM	0.9	
20	VICRYL (polyglactin)	VICRYL	0.9	
21	Polyolefin elastomers	POE	0.9

Detailed information on MPs content for each station is shown in Supplementary Material (Appendix 1) in Microsoft Excel format. Particle distribution by size and colour are provided in Microsoft Excel format in Appendix 2 and Appendix 3, respectively. Detailed results of μ-Raman spectroscopy are presented in Appendix 4.

## 2 Experimental design, materials, and methods

### 2.1 Sediment sampling

Sediment samples were collected at 53 stations in the Baltic Proper (Fig. 1) during six cruises of oceanographic research vessels and two expeditions on small boats in the coastal zone. Ordered by time, the cruises are: (1) boat (July 3, 2015); (2) boat (October 9, 2015); (3) RV NORD (cruise NORD March 30, 2016); (4) RV Professor Shtokman (cruise Sht 131: March 31 - April 5, 2016); (5) RV Professor Shtokman (cruise Sht 132: June 12–15, 2016), (6) RV Akademik Nikolay Strakhov (cruise ANS 32 August 5 - September 10, 2016); (7) RV NORD (cruise NORD October 27, 2016); (8) RV Akademik Nikolay Strakhov (cruise ANS 33 December 24, 2016) (Table 1). The sampling of the upper 5–7 cm of bottom sediments was performed at the depths from 3 m to 215 m using different sampling tools: (i) a hand-operated drag with mouth size of 200 × 100 mm (8 samples), (ii) a Van Veen grab (0.1 m^2^) (24 samples), and (iii) an “Ocean-50” grab (0.25 m^2^) (21 samples). The sampled bottom deposits had different grain sizes ranging from clayey mud to mixed medium or coarse sand and gravel with stones [5]. The mass of an individual sample varied from 0.4 kg to 70 kg. All the samples were stored and transported in a closed metallic bucket or can and were homogenized prior to handling in the laboratory with a steel mixer. The buckets containing samples were stored at room temperature until analysis, and clean stainless-steel spoons were used for removing samples from the bucket.

### 2.2 Methods

#### 2.2.1 Sample preparation

Microplastics were extracted from the sediment samples using the method employed by Ref. [1] with modifications [2,3]. To maximize extraction rates, sediments with high clay content were washed through a sieve cascade (0.333 μm, 174 μm, 174 μm) before the extraction to remove clayey mud fractions, which hampers the extraction process [3]. The sediment retained by the sieves was subjected to flotation (Fig. 2).

In brief, the modified NOAA method consists of the following main steps [2,3]: (1) Multiple MPs extraction from a sediment sample by means of density separation with the ZnCl_2_ solution (specific density 1.6 g mL^−1^), (2) Filtering of supernatant solution above the sediment with the filter funnel, (3) Wet peroxide oxidation on the water bath, (4) Calcite fraction digestion with HCl solution, (5) Filtering with filter funnel, (6) Density separation to detach oxidized organic matter, (7) Filtering with filter funnel, (8) MPs detection with a stereomicroscope, and additionally (9) MPs identification with a Raman spectrometer (Fig. 2).

#### 2.2.2 Analytical techniques

The MP particles were optically analysed and photographed using a stereomicroscope (Micromed MC2 Zoom Digital) with magnification from ×10 to ×40 directly on the filter surface according to recommendations for microscopic determination [6].

All the analysis and detection procedures were performed by the single operator to exclude inter-operator variability. Since plastics particles cannot be fully exactly identified only by visual observation [[7], [8], [9], [10], [11]], μ-Raman spectroscopy was used to verify the result and attain the composition of plastic-like particles [12]. Raman Centaur U (LTD «NanoScanTechnology», Russia) spectrometer was used to obtain plastic spectra [13].

#### 2.2.3 Contamination control and quality analysis

Metal laboratory equipment and glass tableware were used where possible to minimize external contamination. All instruments used during the extraction process were washed with distilled water and dried before the analysis. Cotton lab coats and clothing from non-synthetic materials were used to minimize airborne contamination during samples handling and extraction.

Twelve blank samples were run to assess the level of background contamination according to Ref. [3].

As an additional measure to control the extraction efficiency, artificial reference particles (ARPs) were added to each sample prior to the extraction procedure. Rectangular ARPs with the side dimension of 0.88 ± 0.41 mm (p = 0.05; n = 40) were prepared from a sheet of fluorescent PET 0.46 mm ± 0.02 mm thick (p = 0.05; n = 40). These ARPs, with their artificial shape and characteristic fluorescence, are easily distinguishable from MPs of natural sediments, and provide a clear indication of the quality of the extraction procedure [3].

#### 2.2.4 Classification methods

A visual assessment was performed to identify the shape, size, and colour of MPs according to the physical characteristics of the particles. The extracted MPs were classified into three groups: fragments, films, and fibres according to Ref. [14].

Particle colour was divided into the following categories: transparent, white, green, blue, yellow, red, brown, and black, which is close to categories according to Refs. [8,15]. The blue category included deep blue, light blue, and violet particles. The yellow category also included orange particles. The transparent category included colourless and muddy particles. The red category also included pink and purple particles. The black category included transparent black and grey particles.

The extracted particles were divided into 24 categories using similarity of their visual appearance (shapes, colours), mechanical quality (rigid, soft, elastic, foamed, etc.), and behaviour during a hot-needle test.

#### 2.2.5 μ-Raman spectroscopy verification

The analysis procedure followed [13]. Out of the identified MPs, the core polymer type of some specimens was impossible to identify because of the strong signal induced by synthetic dyes (SD) or strong background fluorescence. Still, the fact of presence of SD was considered as confirmation of synthetic origin of a particle. So, all such specimens were accounted as MPs (for example, Fig. 3). Polymer type and types of synthetic dyes identified using μ-Raman spectroscopy are presented in Table 3. In other cases, the identification by μ-Raman spectroscopy was not possible due to too small particle size or chemical compounds remaining on the surface of a particle. Raman spectra of top 8 typical MPs are presented in Fig. 4.

## References

[1] Masura J., Baker J., Foster G., Arthur C. (2015). Laboratory methods for the analysis of microplastics in the marine environment: recommendations for quantifying synthetic particles in waters and sediments. NOAA Technical Memorandum NOS-OR&R-48.

[2] Zobkov M., Esiukova E. (2017). Microplastics in Baltic bottom sediments: quantification procedures and first results. Mar. Pollut. Bull..

[3] Zobkov M., Esiukova E. (2017). Evaluation of the Munich Plastic sediment separator efficiency in extraction of microplastics from natural marine bottom sediments. Limnol Oceanogr. Methods.

[4] Seifert T., Tauber F., Kayser B. (2001). A high resolution spherical grid topography of the Baltic Sea – revised edition. The Baltic Sea Science Congress.

[5] Wentworth C.K. (1922). A scale of grade and class terms for clastic sediments. J. Geol..

[6] Norén F. (2007). Small Plastic Particles in Coastal Swedish Waters.

[7] Silva A.B., Bastos A.S., Ana S., Justino C.I.L., da Costa J.P., Duarte A.C., Rocha-Santos T.A.P. (2018). Microplastics in the environment: challenges in analytical chemistry - a review. Anal. Chim. Acta.

[8] Yan M., Nie H., Xu K., He Y., Hu Y., Huang Y., Wang J. (2019). Microplastic abundance, distribution and composition in the pearl river along guangzhou city and pearl river estuary, China. Chemosphere.

[9] Löder M.G.J., Gerdts G., Bergmann M., Gutow L., Klages M. (2015). Methodology used for the detection and identification of microplastics—a critical appraisal. Marine Anthropogenic Litter.

[10] Rocha-Santos T., Duarte A.C. (2015). A critical overview of the analytical approaches to the occurrence, the fate and the behavior of microplastics in the environment. TrAC Trends Anal. Chem..

[11] Hidalgo-Ruz V., Gutow L., Thompson R.C., Thiel M. (2012). Microplastics in the marine environment: a review of the methods used for identification and quantification. Environ. Sci. Technol..

[12] Araujo C.F., Nolasco M.M., Ribeiro A.M., Ribeiro-Claro P.J. (2018). Identification of microplastics using Raman spectroscopy: latest developments and future prospects. Water Res..

[13] Zobkov M.B., Esiukova E.E., Zyubin A.Y., Samusev I.G. (2019). Microplastic content variation in water column: the observations with novel sampling tool in stratified Baltic Sea. Mar. Pollut. Bull..

[14] Chubarenko I., Esiukova E., Bagaev A., Isachenko I., Demchenko N., Zobkov M., Efimova I., Bagaeva M., Khatmullina L., Zeng E.Y. (2018). Behavior of microplastics in coastal zones. Microplastic Contamination in Aquatic Environments.

[15] Zhang C., Zhou H., Cui Y., Wang C., Li Y., Zhang D. (2019). Microplastics in offshore sediment in the yellow Sea and east China Sea, China. Environ. Pollut..

