# Supplementary material for: Data on microplastic contamination of the Baltic Sea bottom sediment samples in 2015–2016
Source: Data Brief. 2019 Nov 26;28:104887. doi: 10.1016/j.dib.2019.104887 (PMC6906683; doi:10.1016/j.dib.2019.104887)
Supplement: Multimedia component 1 [file mmc1.zip › Supplementary Material/Appendix 4_Spectra, μ-Raman spectroscopy.docx]

**Spectra (**μ-**Raman spectroscopy)**

| Sample  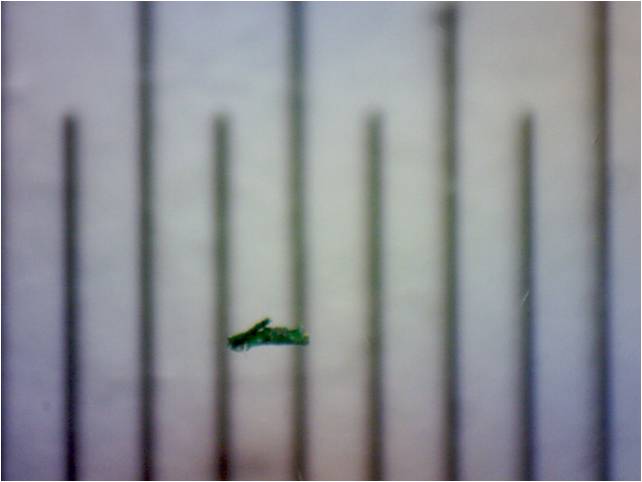 | Name Value  Database Abbreviation RHX  Database Title Raman - Forensic - HORIBA  Record ID 378  Name Hostasol Green G-K  Classification dyestuff  Comments Hoechst  Instrument Name HORIBA LabRAM Infinity-600gr/mm  Raman Laser Power 632.8  Source of Sample LKA Berlin  Source of Spectrum HORIBA Scientific |
| --- | --- |
| 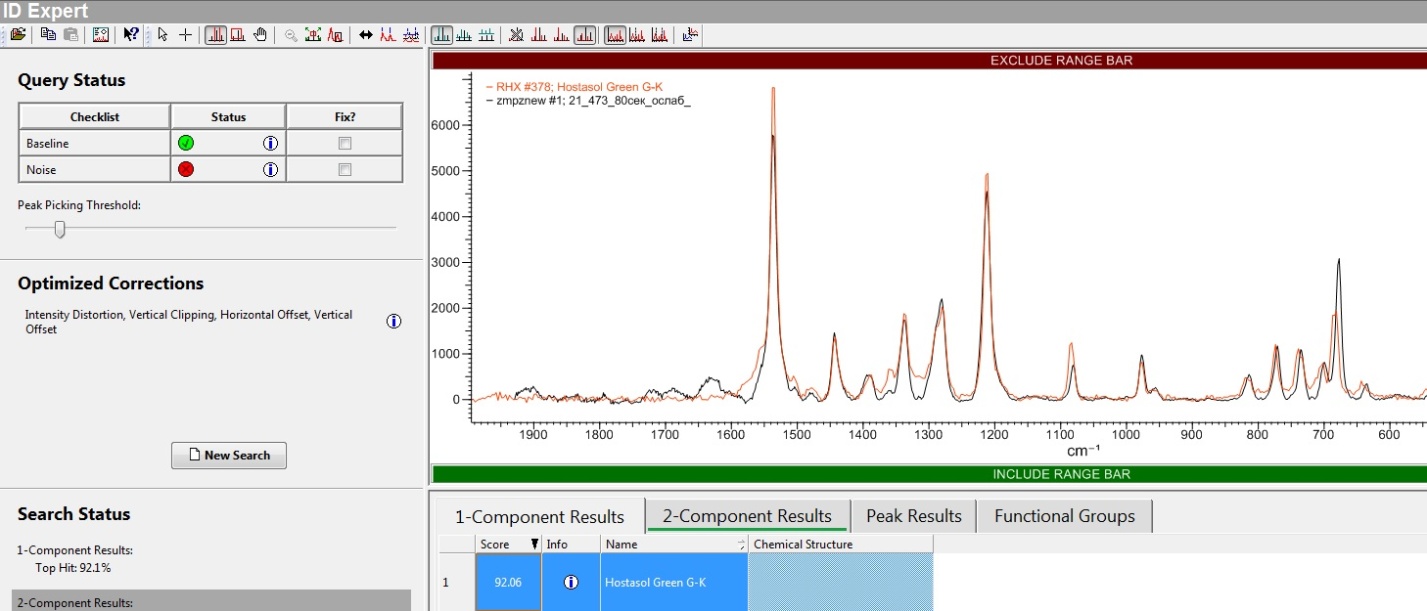 | |

| Sample  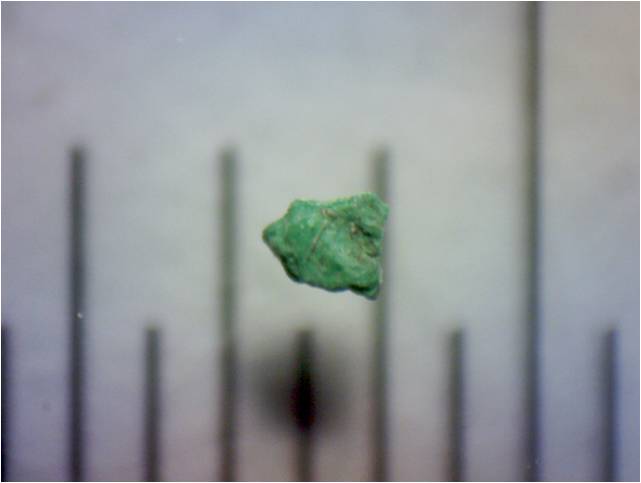 | Name Value  Database Abbreviation RHX  Database Title Raman - Forensic - HORIBA  Record ID 378  Name Hostasol Green G-K  Classification dyestuff  Comments Hoechst  Instrument Name HORIBA LabRAM Infinity-600gr/mm  Raman Laser Power 632.8  Source of Sample LKA Berlin  Source of Spectrum HORIBA Scientific |
| --- | --- |
| 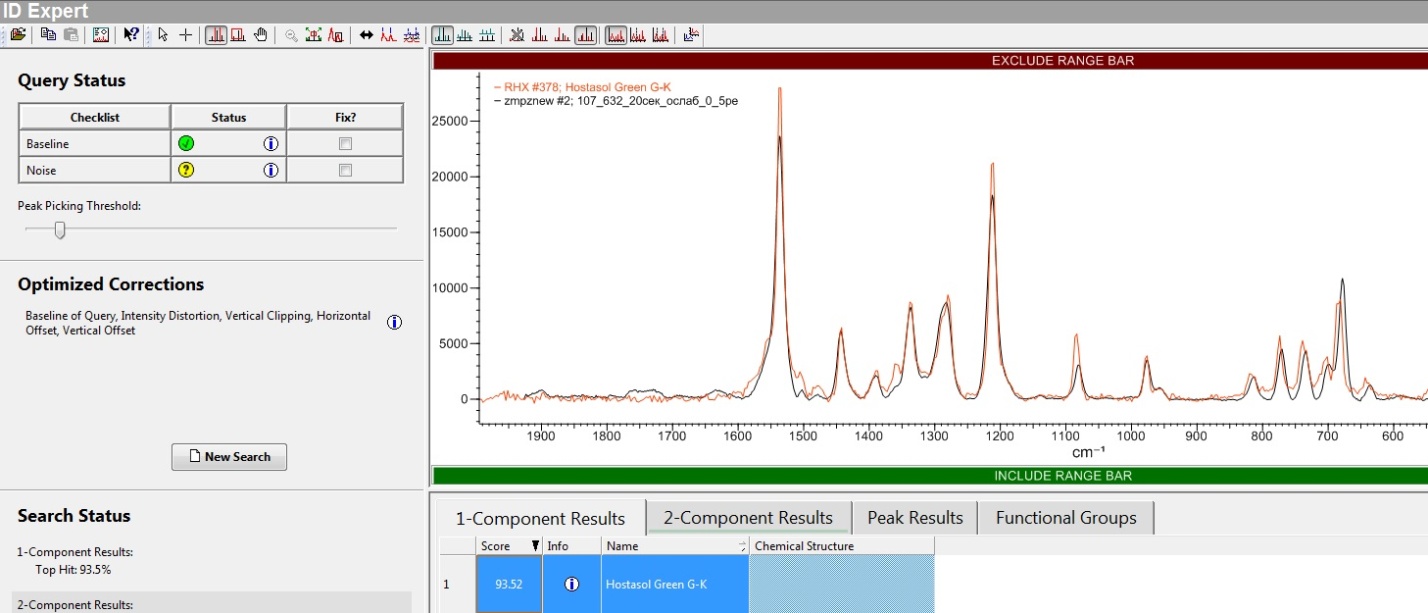 | |

| Sample  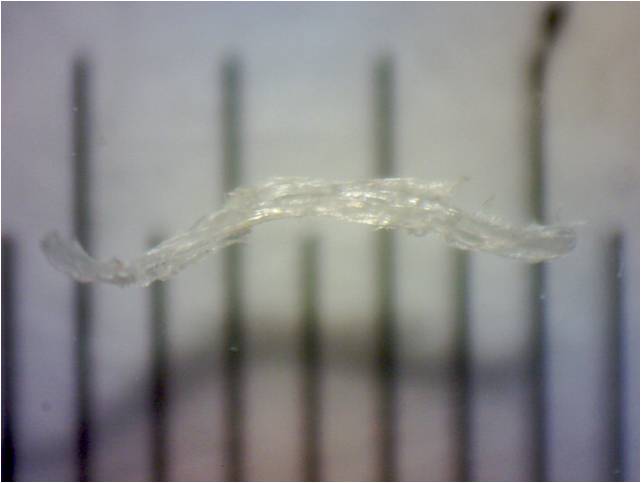 | Name Value  Database Abbreviation RHX  Database Title Raman - Forensic - HORIBA  Record ID 517  Name p-(Ethylene) LD  CAS Registry Number 9002-88-4  Classification polymer  Comments LD=low density; spectrum from branched LDPE  Formula C2H4  Instrument Name HORIBA LabRAM  Raman Laser Power 632.8  Source of Sample Jobin Yvon  Source of Spectrum HORIBA Scientific  Substance Type p-(olefin) |
| --- | --- |
| 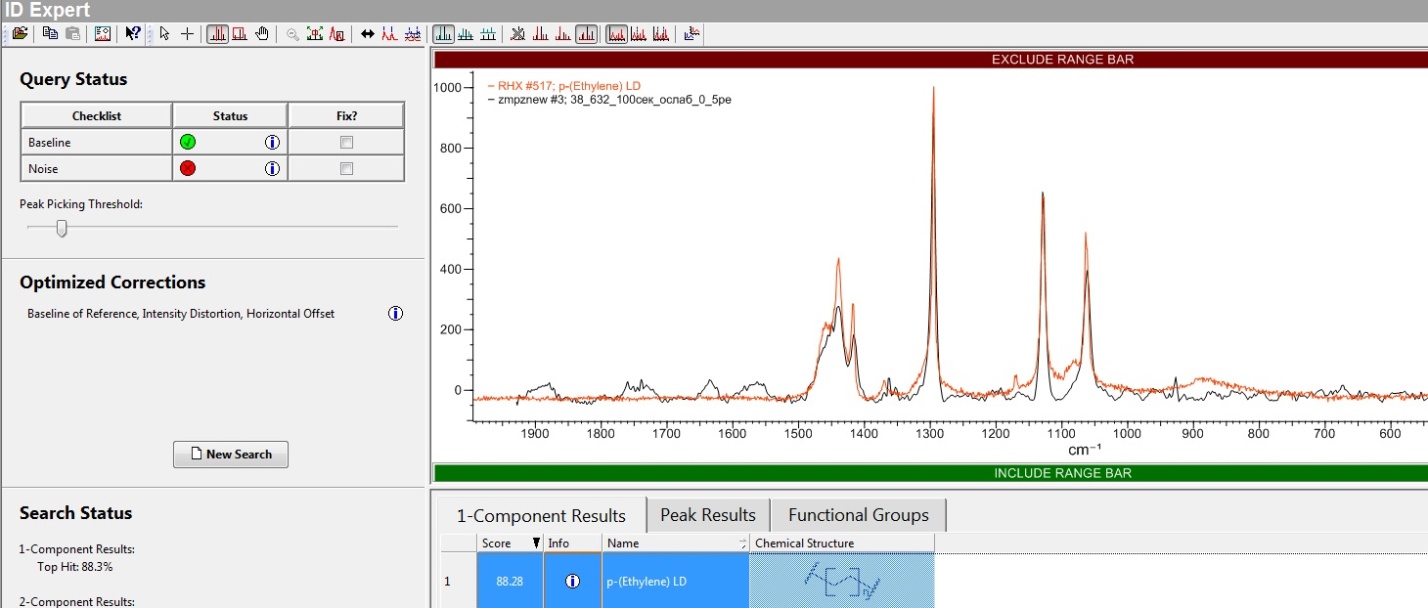 | |

| Sample  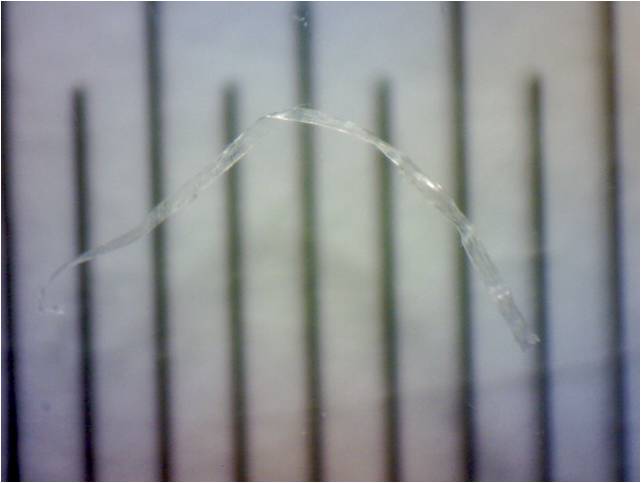 | Name Value  Database Abbreviation RAX  Database Title Raman - Polymers & Processing Chemicals - Bio-Rad Sadtler  Record ID 324  Name Low ethylene RCP  Classification low ethylene random copolymer  Instrument Name Thermo Nicolet FT-Raman 960  Raman Laser Wavelength 1064  Source of Sample ATOFINA Petrochemicals, Inc.  Technique FT-Raman |
| --- | --- |
| 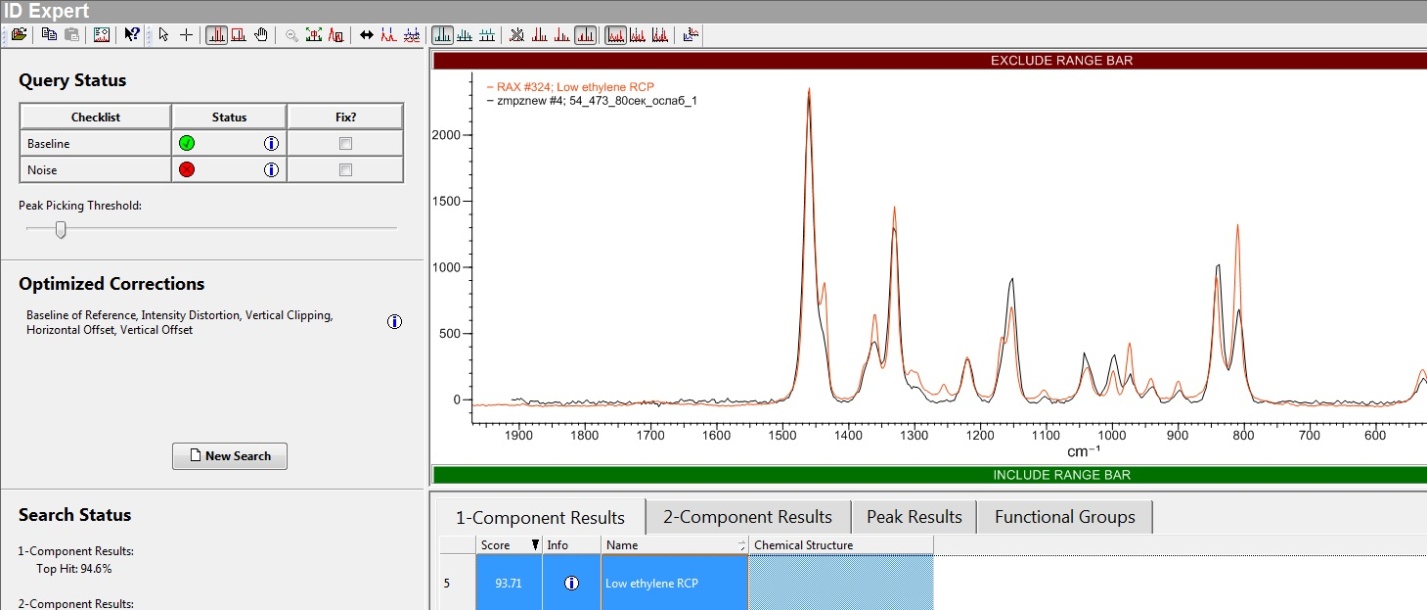 | |

| Sample  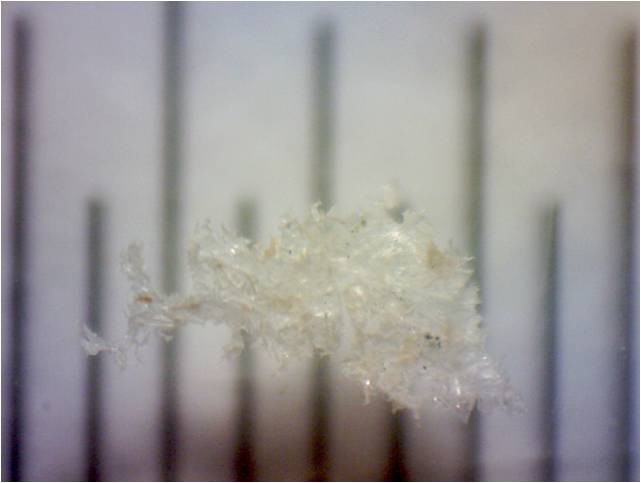 | Composite  Name Value  Database Abbreviation WSARX  Database Title Raman - Aldrich Library of Raman Spectra - Wiley  Record ID 2722  Name Poly(ethylene-co-vinyl acetate), 14 wt.% vinyl acetate  Brand ALDRICH  CAS Registry Number 24937-78-8  Catalog Number 181056  Formula (reported) (CH2CH2)m[CH2CH(OCOCH3)]n  Wiley ID SIAL_RAMAN_002757 |
| --- | --- |
| 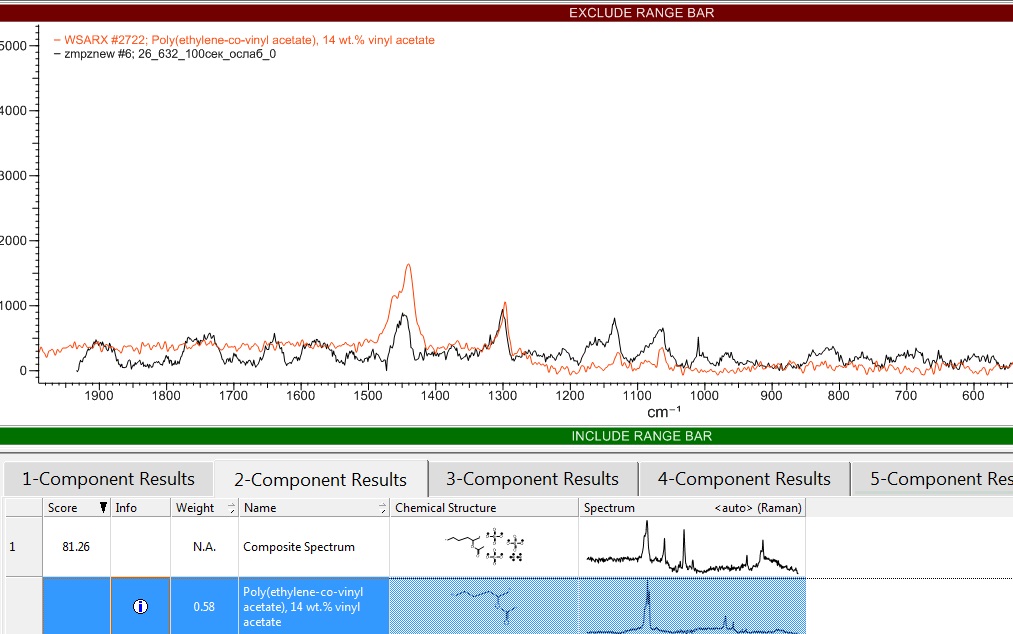 | |

| Sample  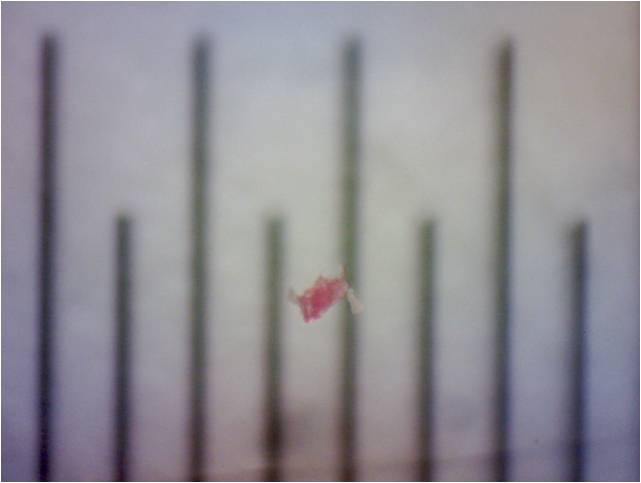 | Name Value  Database Abbreviation RHX  Database Title Raman - Forensic - HORIBA  Record ID 550  Name p-(Propylene)  CAS Registry Number 25085-53-4  Classification polymer  Formula C3H6  Instrument Name HORIBA  Raman Laser Power 632.8  Source of Sample Jobin Yvon  Source of Spectrum HORIBA Scientific  Substance Type p-(olefin) |
| --- | --- |
| 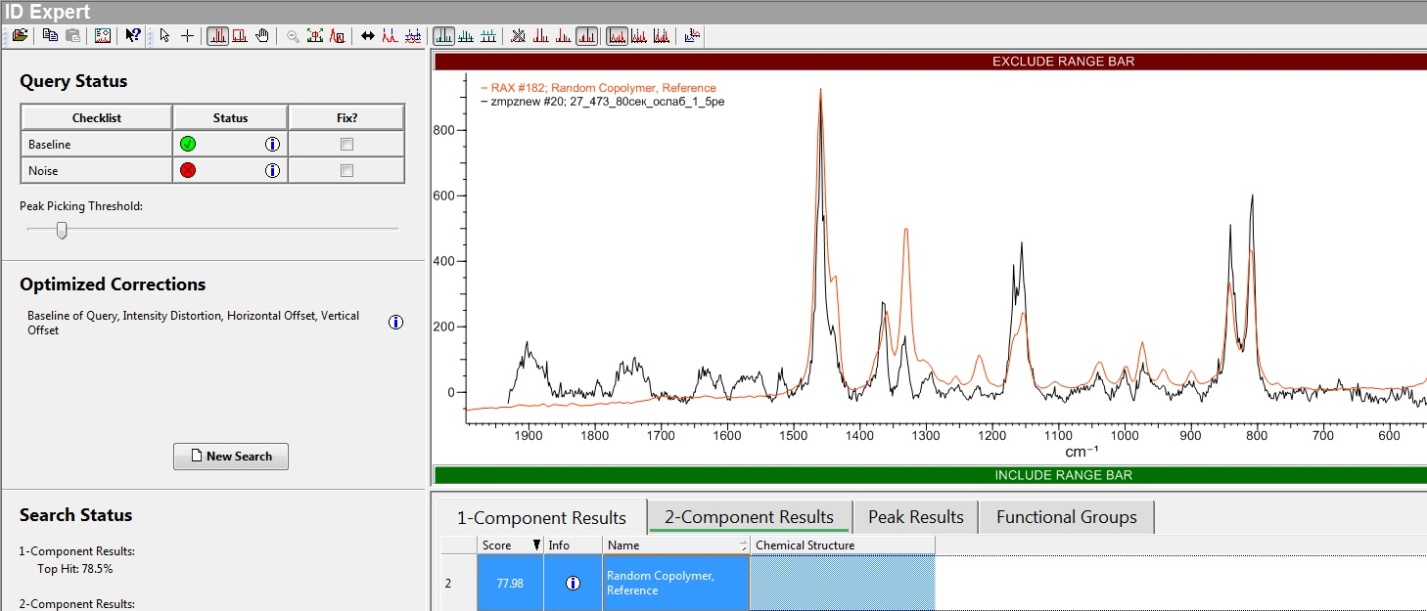 | |

| Sample  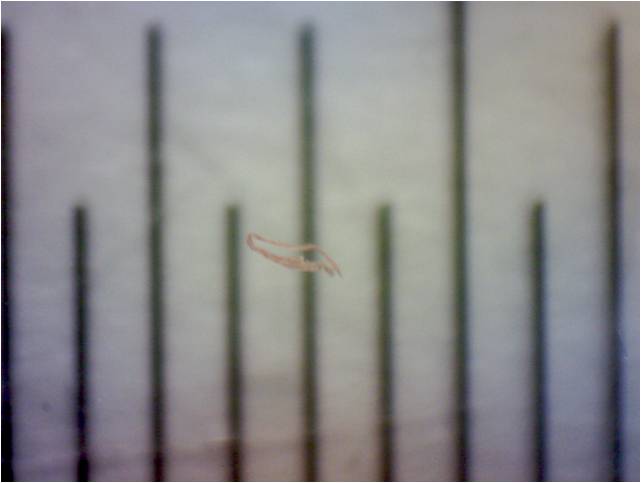 | Name Value  Database Abbreviation RHX  Database Title Raman - Forensic - HORIBA  Record ID 550  Name p-(Propylene)  CAS Registry Number 25085-53-4  Classification polymer  Formula C3H6  Instrument Name HORIBA  Raman Laser Power 632.8  Source of Sample Jobin Yvon  Source of Spectrum HORIBA Scientific  Substance Type p-(olefin) |
| --- | --- |
| 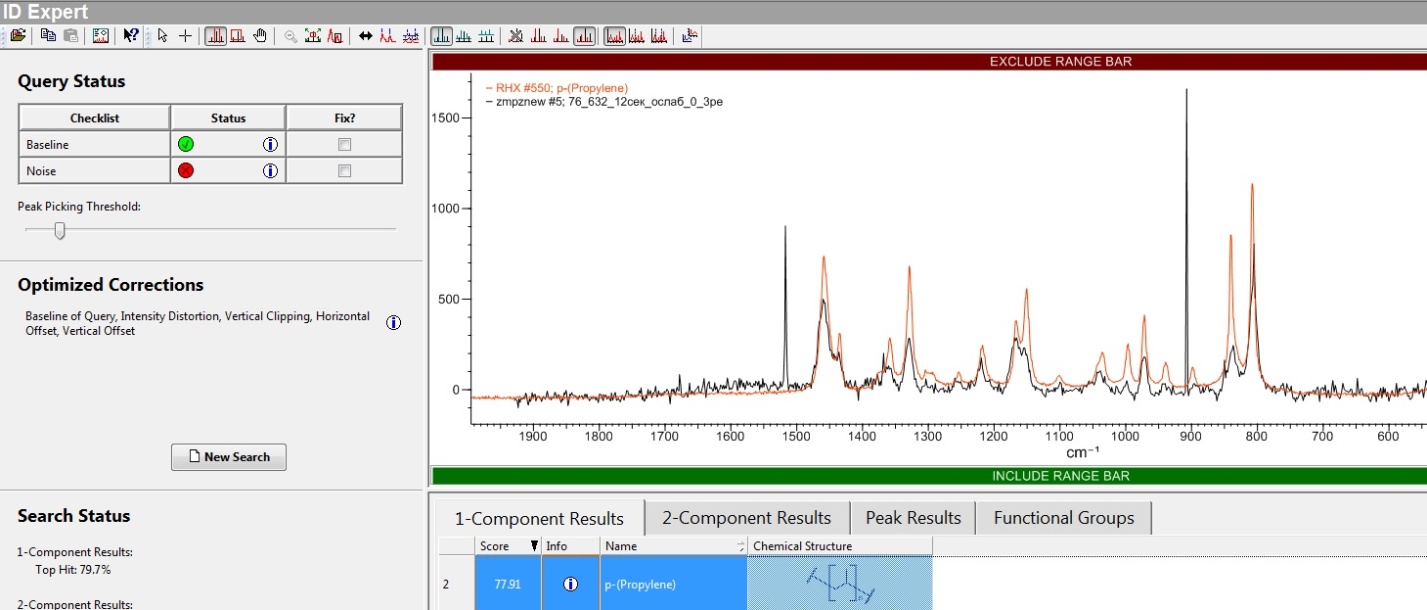 | |

| Sample  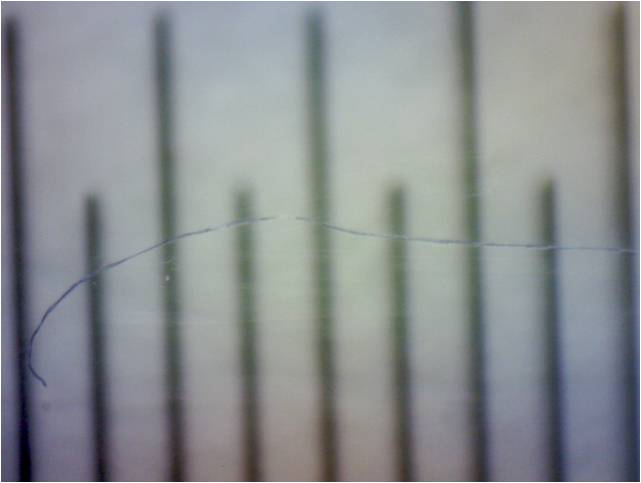 | Name Value  Database Abbreviation QRX  Database Title Raman - Polymers & Monomers (Basic) - Bio-Rad Sadtler  Record ID 403  Name POLY(DIMETHYLPHENYLMETHYLSILOXANE)  Classification SILICONE POLYMERS  Dynamic Viscosity 50 CPS  Formula (C2H6OSi)n + (C7H8OSi)n  Instrument Name Bio-Rad FTS 175C with Raman accessory  Raman Corrections Referenced to internal white light source; Baseline subtracted  Raman Laser Source Nd:YAG  Raman Laser Wavelength 1064  Source of Sample POLYSCIENCES, INC., WARRINGTON, PENNSYLVANIA  Technique FT-Raman |
| --- | --- |
| 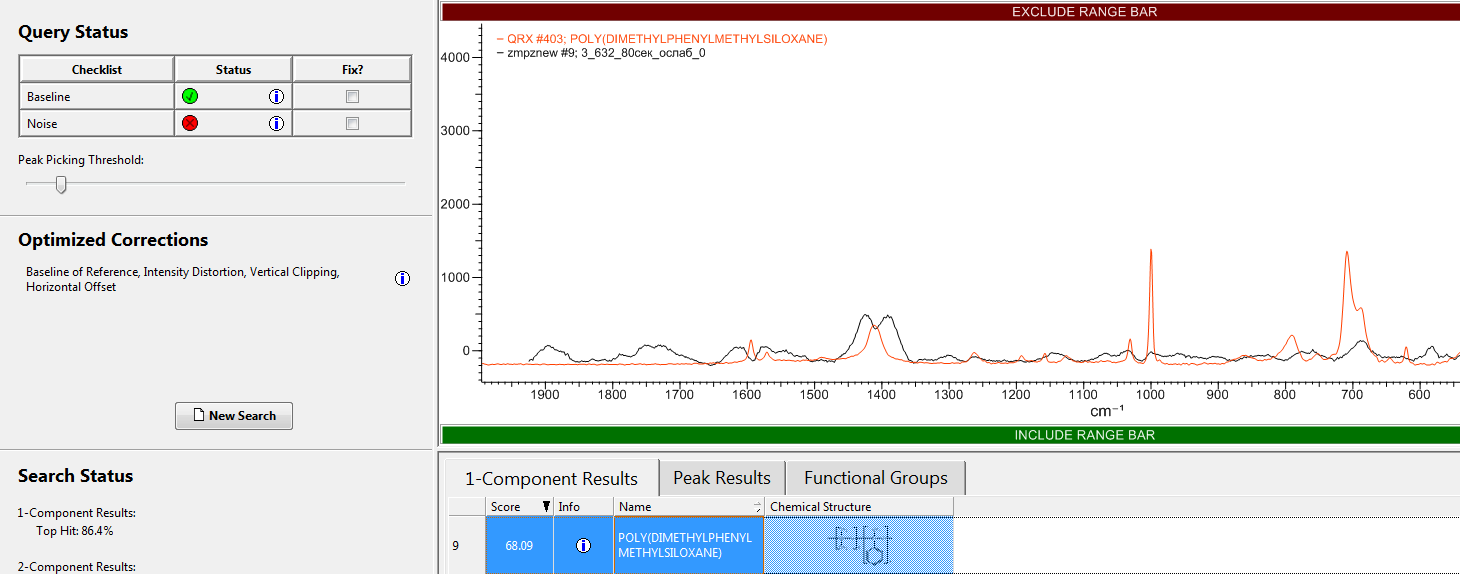 | |

| Sample  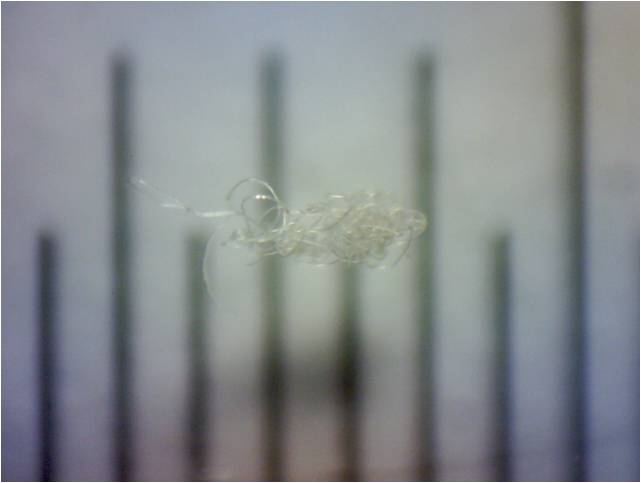 | Name Value  Database Abbreviation WSARX  Database Title Raman - Aldrich Library of Raman Spectra - Wiley  Record ID 2722  Name Poly(ethylene-co-vinyl acetate), 14 wt.% vinyl acetate  Brand ALDRICH  CAS Registry Number 24937-78-8  Catalog Number 181056  Formula (reported) (CH2CH2)m[CH2CH(OCOCH3)]n  Wiley ID SIAL_RAMAN_002757 |
| --- | --- |
| 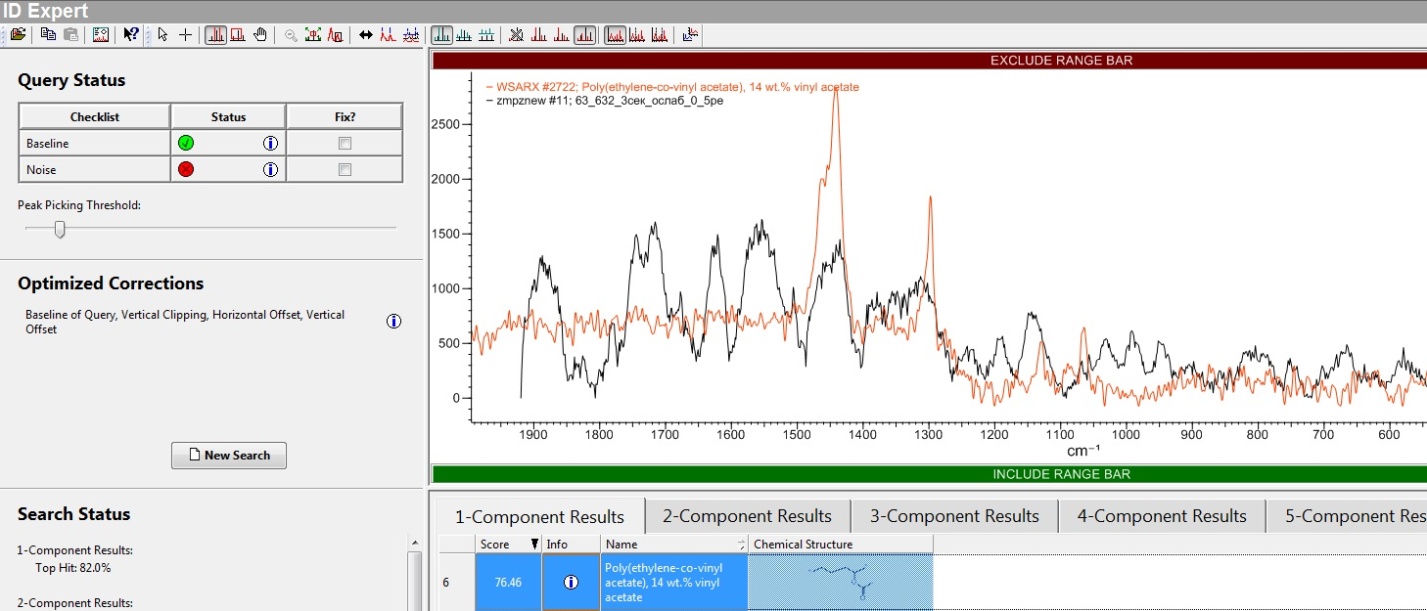 | |

| Sample  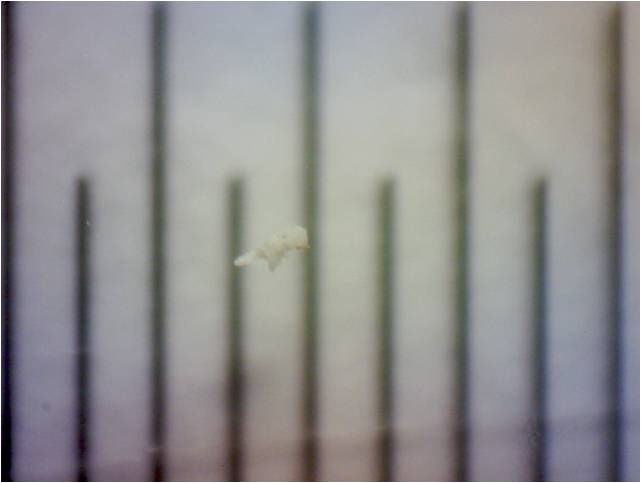 | Name Value  Database Abbreviation QRX  Database Title Raman - Polymers & Monomers (Basic) - Bio-Rad Sadtler  Record ID 909  Name POLYESTER FILM*3000 SERIES  Classification POLYESTERS  Instrument Name Bio-Rad FTS 175C with Raman accessory  Raman Corrections Referenced to internal white light source; Baseline subtracted  Raman Laser Source Nd:YAG  Raman Laser Wavelength 1064  Source of Sample CELANESE CORPORATION, CELANESE ENGINEERING RESINS DIVISION  Synonyms CELANAR POLYESTER FILM*3000 SERIES  Technique FT-Raman |
| --- | --- |
| 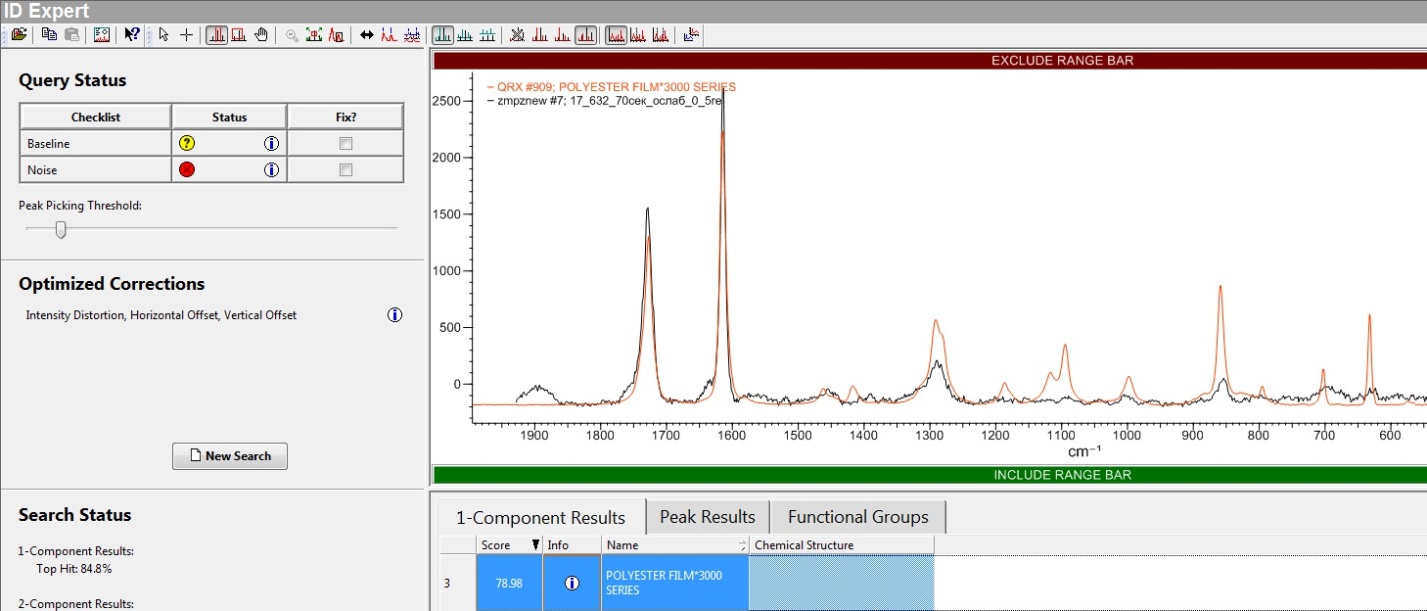 | |

| Sample  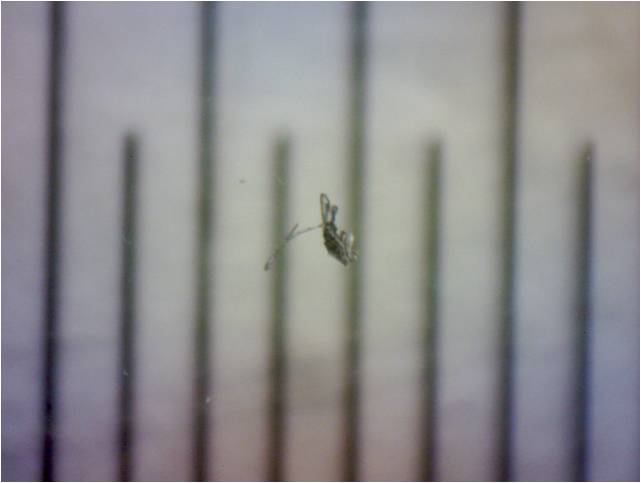 | Name Value  Database Abbreviation RHX  Database Title Raman - Forensic - HORIBA  Record ID 505  Name Cellulose  CAS Registry Number 9004-34-6  Classification polymer  Comments linear  Formula C6H10O5  Instrument Name HORIBA  Raman Laser Power 632.8  Source of Sample Jobin Yvon  Source of Spectrum HORIBA Scientific  Substance Type p-(saccaride); p-(carbohydrate |
| --- | --- |
| 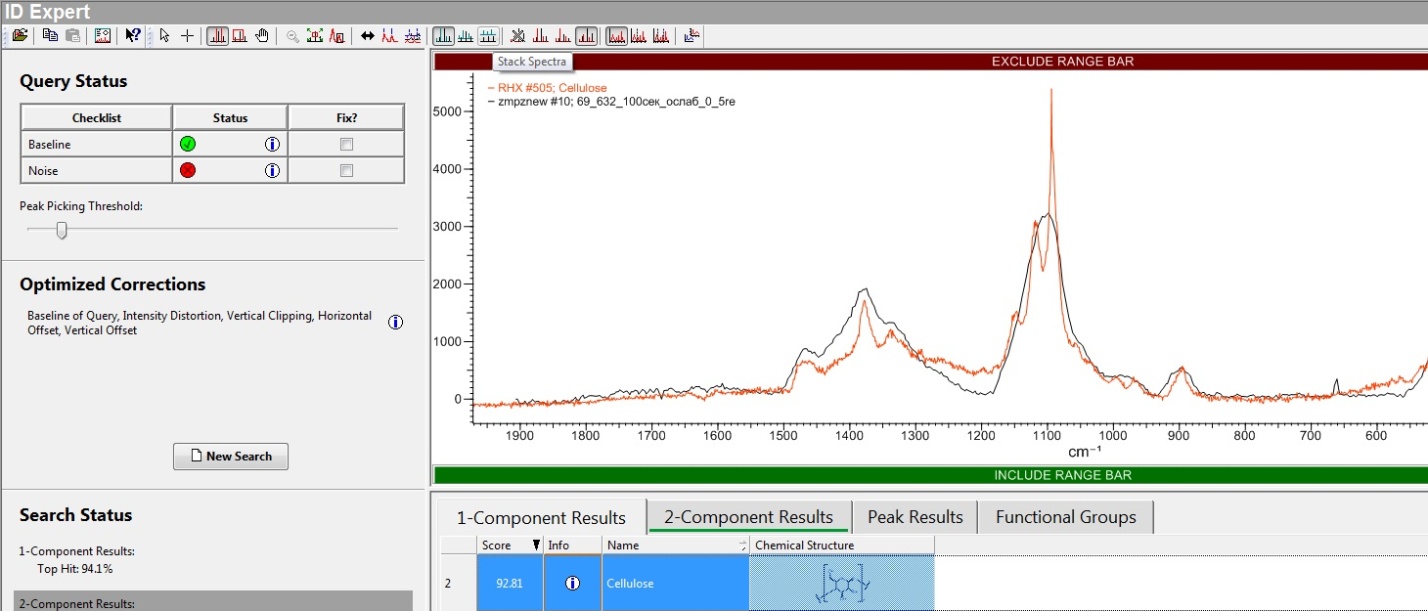 | |

| Sample  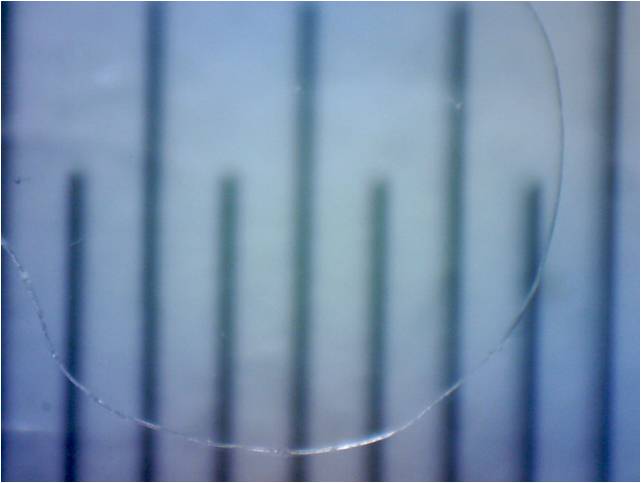 | Name Value  Database Abbreviation RHX  Database Title Raman - Forensic - HORIBA  Record ID 539  Name p-(Ethylene terephthalate)  CAS Registry Number 25038-59-9  Classification polymer  Comments polyester  Formula C10H8O4  Instrument Name HORIBA  Raman Laser Power 632.8  Source of Sample Jobin Yvon  Source of Spectrum HORIBA Scientific  Substance Type p-(ester)  Synonyms Mylar; Terylene; Tergal; Dracon |
| --- | --- |
| 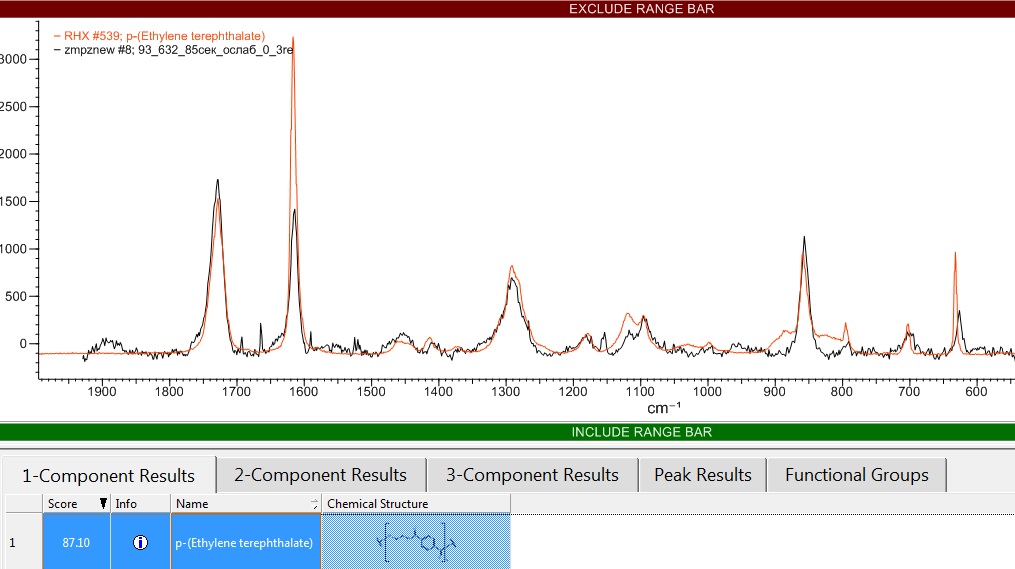 | |

| Sample  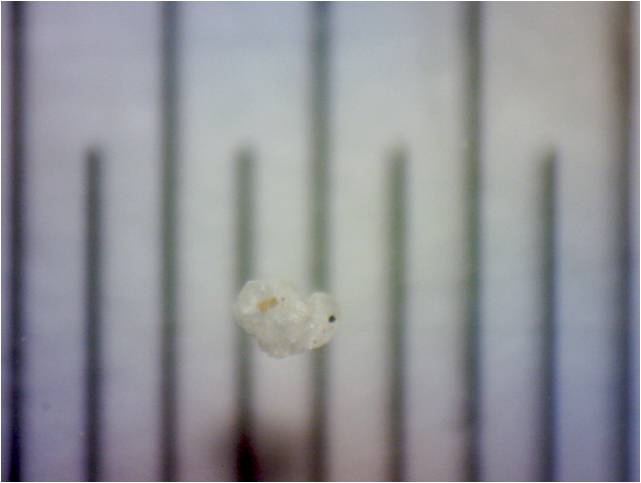 | Name Value  Database Abbreviation RHX  Database Title Raman - Forensic - HORIBA  Record ID 516  Name p-(Ethylene) LD  CAS Registry Number 9002-88-4  Classification polymer  Comments LD=low density  Formula C2H4  Instrument Name HORIBA  Raman Laser Power 632.8  Source of Sample Jobin Yvon  Source of Spectrum HORIBA Scientific  Substance Type p-(olefin) |
| --- | --- |
| 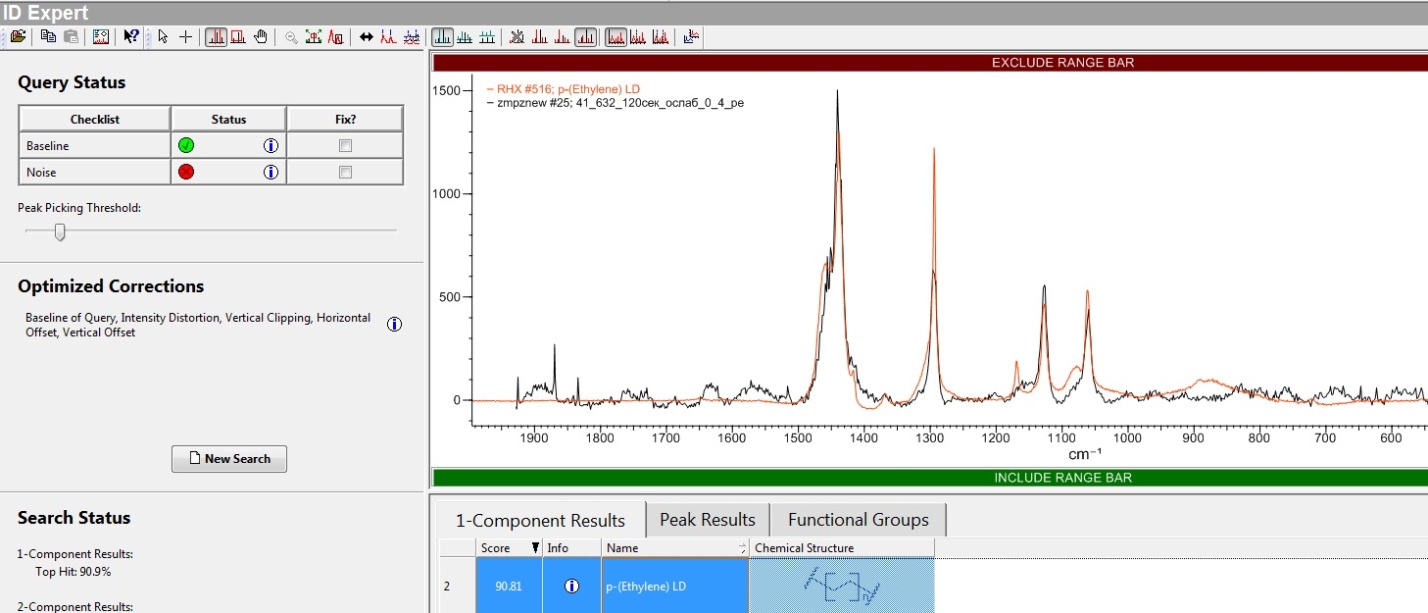 | |

| Sample  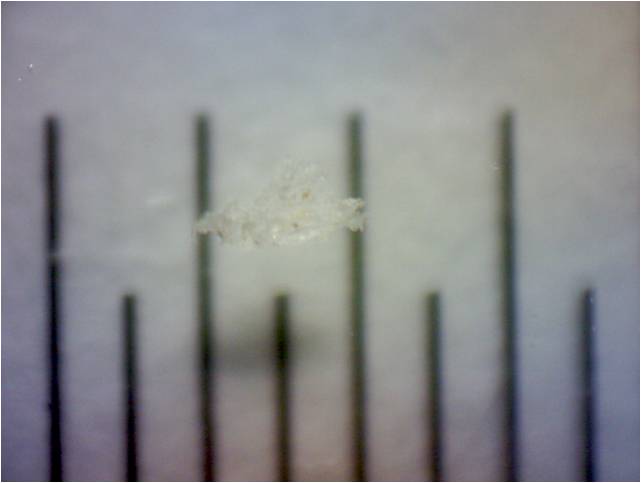 | Name Value  Database Abbreviation QRX  Database Title Raman - Polymers & Monomers (Basic) - Bio-Rad Sadtler  Record ID 487  Name POLYMETHACRYLAMIDE  Classification POLYAMIDES  Formula (C4H7NO)n  Instrument Name Bio-Rad FTS 175C with Raman accessory  Raman Corrections Referenced to internal white light source; Baseline subtracted  Raman Laser Source Nd:YAG  Raman Laser Wavelength 1064  Source of Sample POLYSCIENCES, INC., WARRINGTON, PENNSYLVANIA  Technique FT-Raman |
| --- | --- |
| 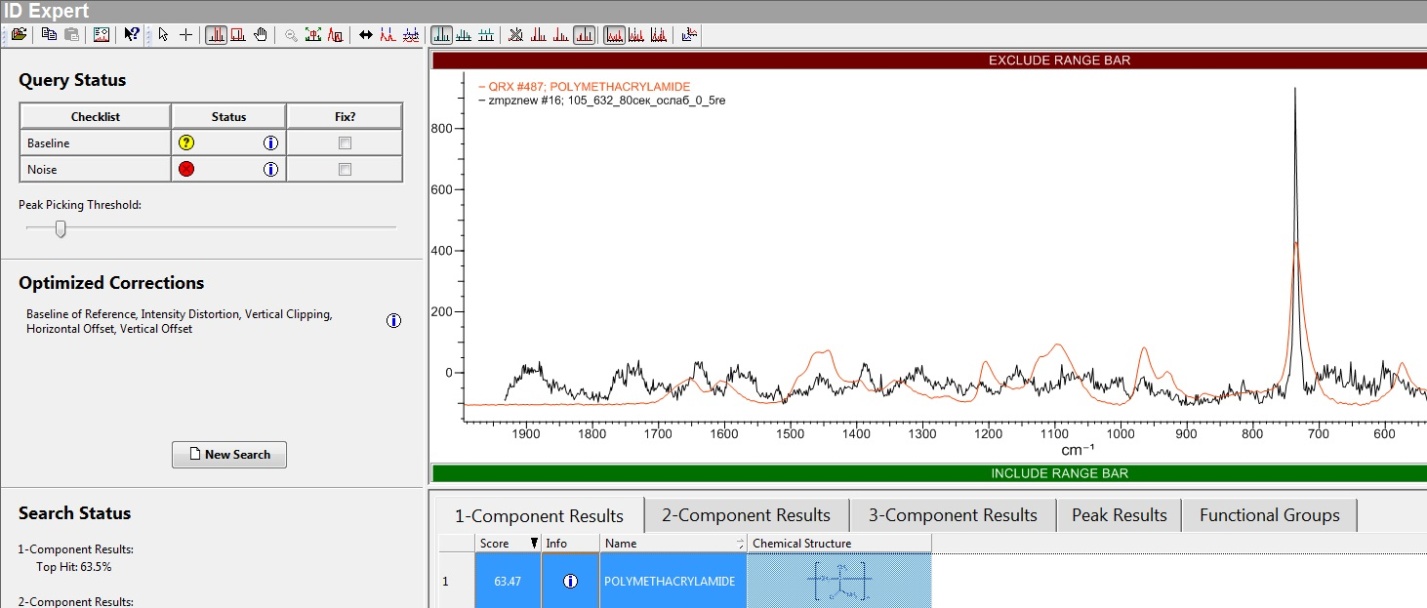 | |

| Sample  polymer blend  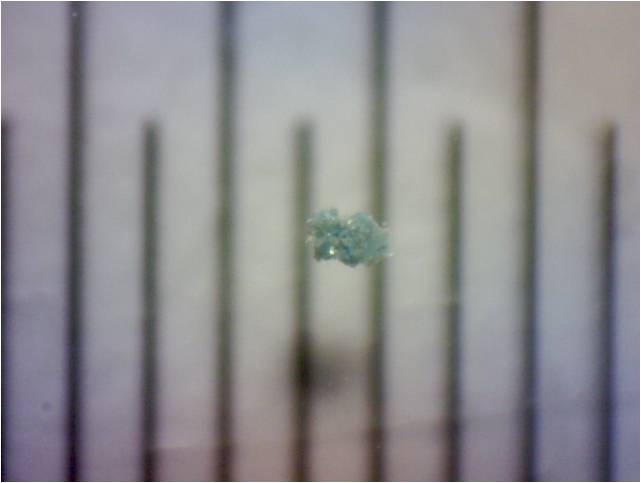 | Name Value  Database Abbreviation RHX  Database Title Raman - Forensic - HORIBA  Record ID 347  Name Cobalt phthalocyanine  CAS Registry Number 3317-67-7  Classification dyestuff  Comments Aldrich 1; CoPc; O.D. 0.3  Formula C32 H16 N8 Co  Instrument Name HORIBA LabRAM Infinity-600gr/mm  Raman Laser Power 632.8  Source of Sample LKA Berlin  Source of Spectrum HORIBA Scientific  Synonyms C.I. Generic name: Pigment Blue 75; Pigment Blue 75; C.I. 74160:2  Name Value  Database Abbreviation QRX  Database Title Raman - Polymers & Monomers (Basic) - Bio-Rad Sadtler  Record ID 1598  Name POLY(PROPYLENE), AMORPHOUS*LOW MOLECULAR WEIGHT  Classification POLYPROPYLENES  Density (Specific Gravity)= (15C) 0.8544  Dynamic Viscosity (99C) 3150 SSU  Flash Point (COC) 237C  Instrument Name Bio-Rad FTS 175C with Raman accessory  Mol.Weight 3000  Raman Corrections Referenced to internal white light source; Baseline subtracted  Raman Laser Source Nd:YAG  Raman Laser Wavelength 1064  Source of Sample CROWLEY TAR PRODUCTS COMPANY  Synonyms POLYPOL 19*LOW MOLECULAR WEIGHT  Technique FT-Raman  Name Value  Database Abbreviation WSARX  Database Title Raman - Aldrich Library of Raman Spectra - Wiley  Record ID 1949  Name o-Phenylene phosphorochloridite  Brand ALDRICH  CAS Registry Number 1641-40-3  Catalog Number 155764  Exact mass 173.963743591  Formula C6H4ClO2P  Formula (reported) C6H4ClO2P  InChI Key YUJYEGDMJZHLMY-UHFFFAOYSA-N  Mol.Weight 174.522 g/mol  Purity 97%  Synonyms 2-Chloro-1,3,2-benzodioxaphosphole; o-Phenylene chlorophosphite; Chloro(1,2-phenylenedioxy)phosphine; Cyclic o-phenylene phosphorochloridite; Phosphorochloridous acid orthophenylene ester; Pyrocatechol phosphoryl chloride  Wiley ID SIAL_RAMAN_001977  Name Value  Database Abbreviation WSARX  Database Title Raman - Aldrich Library of Raman Spectra - Wiley  Record ID 2535  Name Hypophosphorous acid-D3 solution, 50 wt.% in D2O  Brand ALDRICH  CAS Registry Number 57583-56-9  Catalog Number 176680  Exact mass 69.00589609  Formula D3O2P  Formula (reported) D3O2P  InChI Key ACVYVLVWPXVTIT-DZCFLQKHSA-N  Mol.Weight 69.0149 g/mol  Purity 99+ ATOM %  Wiley ID SIAL_RAMAN_002570  Name Value  Database Abbreviation RMX  Database Title Raman - Minerals - HORIBA  Record ID 415  Name Richterite  Classification inosilicate; amphibole group; hydroxide  Comments Wilberforce, Ontario, Canada; laser beam _I_ face 010; con.H  Formula (Na,K)2Ca(Mg,Mn)5Si8O22(OH)2  Instrument Name HORIBA  Raman Laser Power 785  Source of Sample Caltech CIT#8019  Source of Spectrum HORIBA Scientific |
| --- | --- |
| 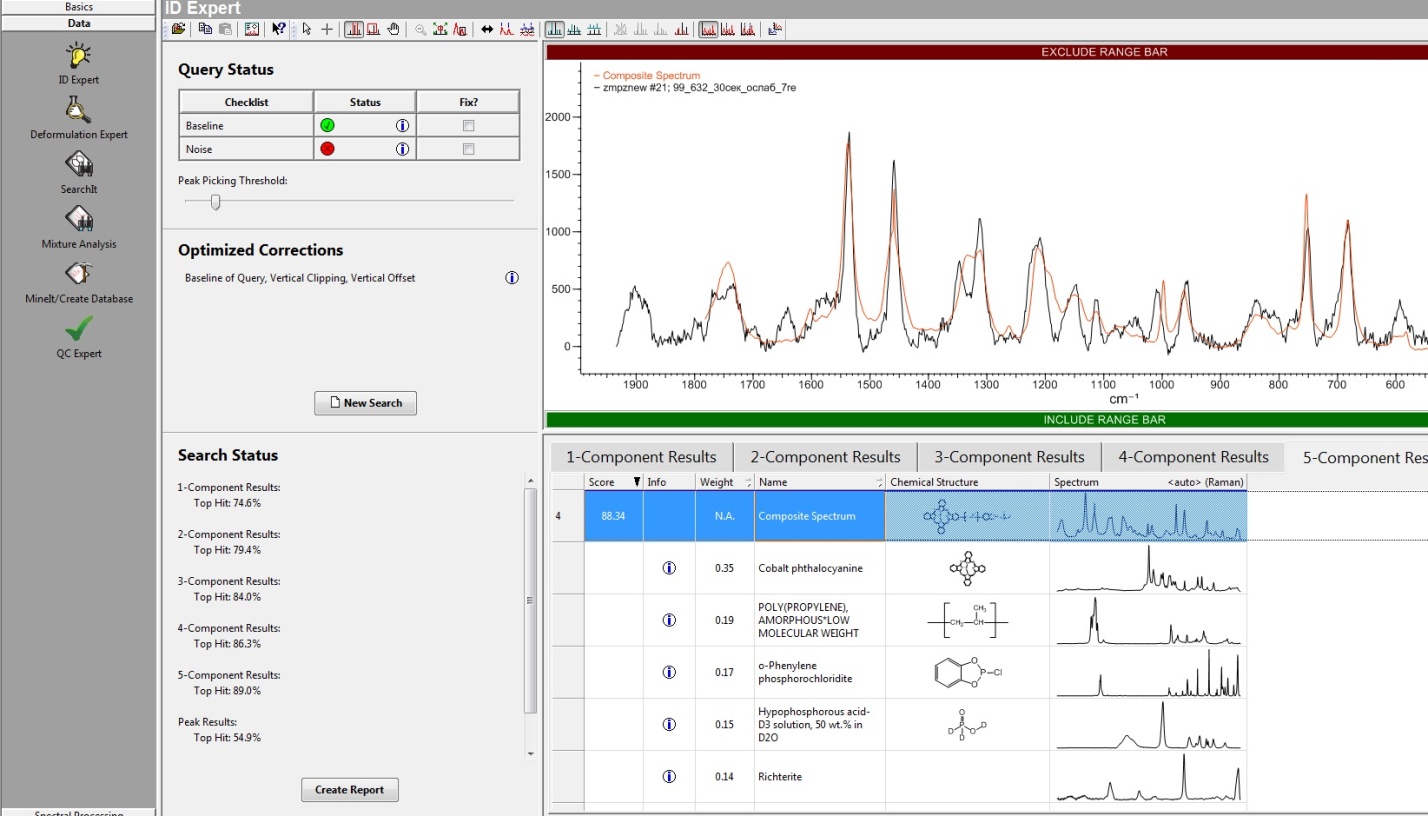 | |

| Sample  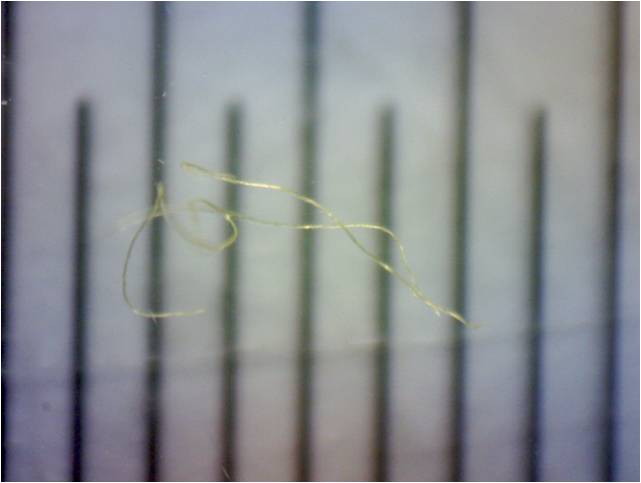 | Name Value  Database Abbreviation RLX  Database Title Raman - Biomaterials - HORIBA  Record ID 73  Name Polyglactin  Comments synthetic absorbable surgical suture  Formula C9H18O4  Instrument Name HORIBA  Mol.Weight 190.240 g/mol  Occurrence rare  Origin Stone No: 30534  Other Properties Risk Factor=Urinary infection can cause the formation of precipitations on the not absorbed material  Raman Laser Power 632.8  Source of Sample Cristal Laboratory Paris + Jobin Yvon, France  Source of Spectrum HORIBA Scientific  Synonyms Copolymer of 90% glycolide + 10% lactide |
| --- | --- |
| 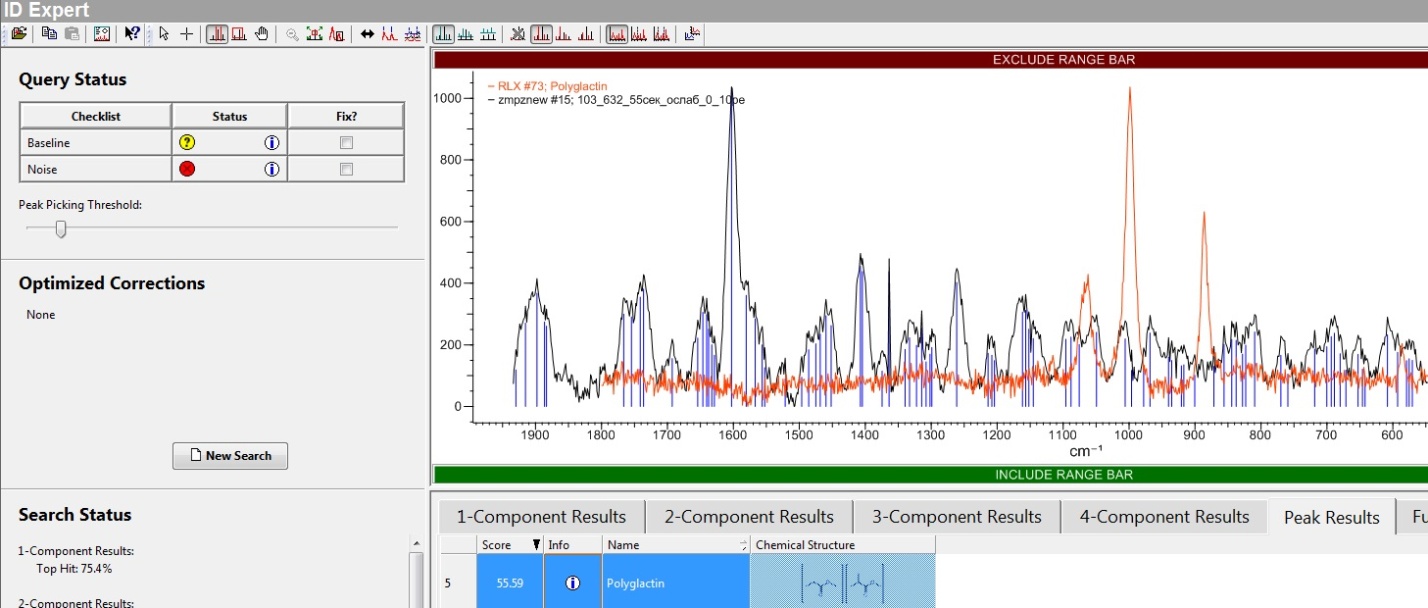 | |
